# Platelet membrane camouflaged AIEgen‐mediated photodynamic therapy improves the effectiveness of anti‐PD‐L1 immunotherapy in large‐burden tumors

**DOI:** 10.1002/btm2.10417

**Published:** 2022-10-06

**Authors:** Jun Dai, Meng Wu, Yating Xu, Hongming Yao, Xiaoding Lou, Yuning Hong, Jian Zhou, Fan Xia, Shixuan Wang

**Affiliations:** ^1^ Department of Obstetrics and Gynecology, Tongji Hospital, Tongji Medical College Huazhong University of Science and Technology Wuhan China; ^2^ College of Material, Chemistry and Chemical Engineering Hangzhou Normal University Hangzhou China; ^3^ State Key Laboratory of Biogeology and Environmental Geology, Engineering Research Center of Nano‐Geomaterials of Ministry of Education, Faculty of Materials Science and Chemistry China University of Geosciences Wuhan China; ^4^ Department of Biochemistry and Chemistry, La Trobe Institute for Molecular Science La Trobe University Melbourne Victoria Australia

**Keywords:** aggregation‐induced emission, immunotherapy, large‐burden tumors, PD‐L1, photodynamic therapy, platelet membranes

## Abstract

Although immunotherapy has achieved recent clinical success in antitumor therapy, it is less effective for solid tumors with large burdens. To overcome this challenge, herein, we report a new strategy based on platelet membrane‐camouflaged aggregation‐induced emission (AIE) luminogen (Plt‐M@P) combined with the anti‐programmed death ligand 1 (anti‐PD‐L1) for tumoral photodynamic‐immunotherapy. Plt‐M@P is prepared by using poly lactic‐co‐glycolic acid (PLGA)/PF3‐PPh_3_ complex as a nanocore, and then by co‐extrusion with platelet membranes. PF3‐PPh_3_ is an AIE‐active conjugated polyelectrolyte with photosensitizing capability for photodynamic therapy (PDT). Plt‐M@P exhibits superior tumor targeting capacity in vivo. When applied in small tumor‐bearing (~40 mm^3^) mice, Plt‐M@P‐mediated PDT significantly inhibits tumor growth. In tumor models with large burdens (~200 mm^3^), using Plt‐M@P‐mediated PDT or anti‐PD‐L1 alone is less effective, but the combination of both is effective in inhibiting tumor growth. Importantly, this combination therapy has good biocompatibility, as demonstrated by the absence of damage to the major organs, especially the reproductive system. In conclusion, we show that Plt‐M@P‐mediated PDT can improve anti‐PD‐L1 immunotherapy by enhancing antitumor effects, providing a promising strategy for the treatment of tumors with large burdens.

## 1 INTRODUCTION

Immunotherapy has achieved accelerated development in particular for clinical cancer treatment over the past few years, among which chimeric antigen receptor‐modified T (CAR‐T) cell therapy and the inhibitors of immune checkpoints are the most well‐known.^1^, ^2^, ^3^ The axis of programmed cell death protein 1 (PD‐1) and programmed death ligand 1 (PD‐L1) plays a critical role in immune homeostasis and the application of immune checkpoint inhibitors to block PD‐1/PD‐L1 interaction shows recent clinical success.^4^, ^5^, ^6^, ^7^ Early clinical trials demonstrated that immunotherapies could achieve long‐lasting remissions in patients with hematologic malignancies.^8^, ^9^, ^10^ However, treatment of solid tumors with immunotherapies has yielded limited therapeutic benefits to date.^11^, ^12^, ^13^ Although an active immunotherapy strategy is usually effective for small tumor burdens, it is much less so for large tumor burdens.^14^, ^15^, ^16^ The key immunotherapies barriers within solid tumors, especially in large tumors, could be attributed to the immunosuppressive tumor microenvironment.^17^ Preclinical data support the findings that large tumors are more immunosuppressive compared to small tumors.^16^ The immunosuppressive state of the tumor microenvironment is enforced via immune cell subsets recruited or induced, such as T cell, dendritic cells, and natural killer cells.^18^ It is suggested that targeting regulating immune cell populations may help convert the tumor microenvironment of large tumors to more closely resemble the immune infiltrate of small tumors.^16^ Hence, strategies to remove these obstacles in large‐burden tumors are primarily focused on the combination of different anticancer therapies, aiming to make the tumors more vulnerable to immunotherapy.^19^, ^20^, ^21^

Photodynamic therapy (PDT) is a clinically used, minimally invasive therapeutic method.^22^, ^23^, ^24^, ^25^ The key element of PDT is the photosensitizer which is activated by irradiation with a specific wavelength of light.^26^, ^27^, ^28^ Upon irradiation, photosensitizer generates highly cytotoxic reactive oxygen species (ROS) to cause oxidative stress‐induced tumor cell death.^29^, ^30^ While traditional photosensitizers, such as porphyrin, phthalocyanine, and analogs, adopt large flat disc‐like structures, are inclined to aggregate and experience strong intermolecular π−π stacking interactions. The excited states of such aggregates often decay to the ground state via nonradiative channels, suffering from aggregation caused quench (ACQ) effect which leads to the reduction of ROS generation efficiency.^31^, ^32^ In addition, both Ce6 and ICG are typical ACQ photosensitizers, and the higher the degree of their aggregation, the lower the yield of resulting ROS instead.^31^, ^33^, ^34^, ^35^ The emergence of the photosensitizer with aggregation‐induced emission (AIE) properties can overcome those challenges. While AIE photosensitizers stay in the aggregate state, the nonradiative decay processes are inhibited, and the excited state can thus be harvested for emission as well as ROS generation, exhibiting superior photostability and photosensitizing capacity.^36^, ^37^, ^38^, ^39^, ^40^ Extensive evidences have been found, at the animal level, that AIE photosensitizers have good antitumor effects and opened up many opportunities with great potential for further employing PDT for tumor treatment in a clinical setting.^41^, ^42^, ^43^, ^44^ Hence, it is a pressing issue today to develop high‐performance AIE photosensitizers to fulfill the need of the area. Recent studies reported that small tumors responded well to a single round of PDT but large tumors showed lower response to the same treatment.^45^, ^46^, ^47^, ^48^ This may be due to the limited penetration depth of light PDT and thus low inhibition efficiency for large‐burden tumors.^48^ In addition, the severe hypoxic state and more complex tumor microenvironment in large‐burden tumors also result in discounted efficiency of PDT.^49^ In addition to local tumor ablation, PDT can increase tumor immunogenicity by inducing tumor‐associated antigens exposure, which improves the presentation of tumor‐associated antigens and activation of T lymphocytes.^50^, ^51^, ^52^ Furthermore, PDT enhances the expression of a number of pro‐inflammatory cytokines, including interleukin‐1 (IL‐1), interleukin‐6 (IL‐6), interleukin‐12 (IL‐12), tumor necrosis factor‐α (TNF‐α), and interferon‐γ (IFN‐γ).^50^, ^53^ PDT has multiple effects on improving the intra‐tumor microenvironment and has been demonstrated to improve the treatment outcome of immunotherapies on mice.^54^, ^55^, ^56^, ^57^, ^58^, ^59^ Marrache et al. encapsulated a photosensitizer, zinc phthalocyanine, with CpG to enhance pro‐inflammatory cytokines release, dendritic cells maturation, and activation for metastatic breast cancer.^56^ Our recent study also showed the combination of the efficient PDT with immunologic adjuvants Poly(I:C) for synergistic activation of the immune system for effective tumor treatment.^57^ With these prior successes, we are intrigued to explore whether PDT can improve the immunosuppressive microenvironment and thus enhance the efficacy of immunotherapy against large‐burden tumors.

The major adverse effects of PDT are photosensitivity reactions, including local skin redness, oozing, and hyperpigmentation. To reduce the occurrence of these adverse reactions, reducing the accumulation of photosensitizer at non‐tumor sites is an effective means.^51^, ^60^ In recent years, nanoparticle (NP) drug carrier research has demonstrated to be exceptional to enhance PDT in the treatment of cancer via enhanced drug delivery mechanisms.^61^ Cell membranes, including red blood cells, platelets, immune cells, stem cells, and cancer cells, have recently emerged as new sources of materials for drug delivery systems because of their enhanced biocompatibility, low immunogenicity, and active targeting abilities.^62^, ^63^ Among these materials, platelets have drawn research interest in drug delivery because of their capability to target specific sites and escape the immune system. Platelet membrane also has the capacity to evade phagocytosis while in blood circulation. In addition, platelet membranes express P‐selectin, a cell adhesion protein that can bind to CD44 receptors overexpressed in cancer cells, and thus, these platelet membrane‐coated NPs showed greater uptake by tumor cells in vitro than plain NPs.^64^ Similarly, platelet membrane‐coated NPs exhibited a greater accumulation in tumor sites and exerted enhanced antitumor efficacy in vivo.^65^ Recently, platelet membranes have also been used to coat NPs for enhanced cancer immunotherapy.^66^, ^67^ Based on these advantages of platelet membrane, platelet membrane‐coated NPs hold promise to improve tumor microenvironment in large‐burden tumors and make them more vulnerable to immunotherapy. Therefore, we envisioned that using platelet membrane‐based drug delivery systems can improve the targeting of the AIE photosensitizer, so as to achieve desired therapeutic effects with minimal side‐effects.

**FIGURE 1:**
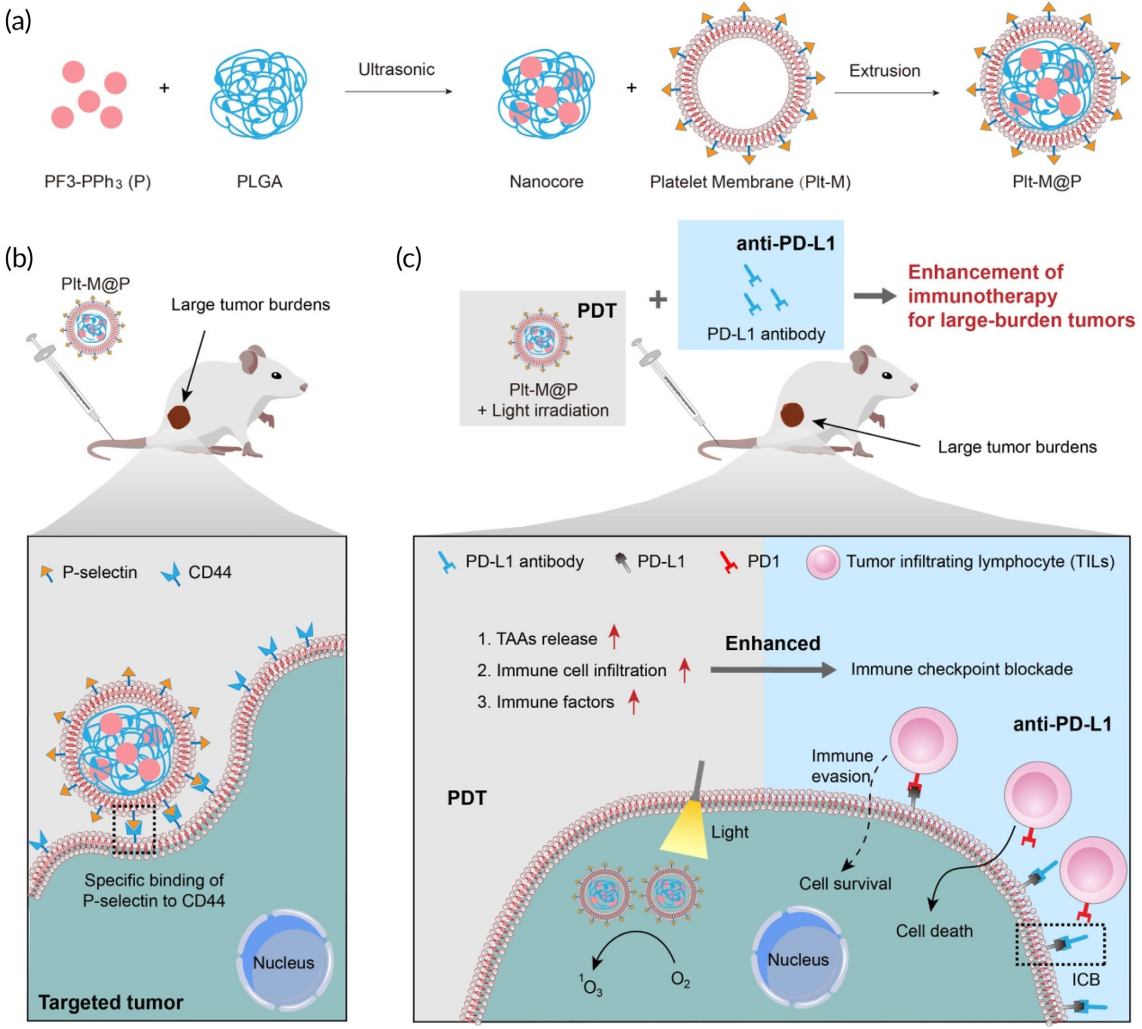
Schematic diagram of preparation and operation of Plt‐M@P. (a) The preparation processes of Plt‐M@P. (b) Plt‐M@P improves tumor targeting by ligand–receptor model. The ligand is p‐selectin on the surface of the NPs and the receptor is CD44 on the tumor cell membrane. (c) Plt‐M@P‐mediated PDT recovers the failure of immune checkpoint blockade in models with large tumor burdens. NP, nanoparticle; PDT, photodynamic therapy.

In this study, we design and synthesize a novel AIE photosensitizing polymer, named PF3‐PPh_3_. Then, PF3‐PPh_3_ (P) and platelet membranes (Plt‐M) are co‐extruded to construct Plt‐M@P NPs (Figure 1a). We demonstrate that Plt‐M@P NPs are able to specifically target CD44 receptors on tumor cells via P‐selectin on platelet membranes,^66^, ^67^, ^68^, ^69^ thus enhancing the accumulation of NPs in tumors (Figure 1b). For small tumors, Plt‐M@P‐mediated PDT is found to effectively inhibit tumor growth. However, for tumors with large burdens, the treatment of PDT mediated by Plt‐M@P or anti‐PD‐L1 alone is less effective. However, the combination therapy of PDT and anti‐PD‐L1 can modulate the immune tumor microenvironment of large tumors, making them more vulnerable to immune response (Figure 1c). PDT combined with anti‐PD‐L1 contributes to the robust and persistent antitumor immunity in large established tumors, which provides a promising strategy for the treatment of large tumor burdens.

## 2 EXPERIMENTAL SECTION

### 2.1 Materials and Instruments

Tetrahydrofuran (THF) was distilled from sodium benzophenone ketyl under dry nitrogen immediately before use. Chemicals and reagents were purchased from Sigma‐Aldrich and J&K Scientific Ltd. ^1^H, ^13^C, and ^31^P nuclear magnetic resonance (NMR) spectra of small molecules, polymers, and polyelectrolytes were measured on a Bruker AV 500 spectrometer in deuterated solvents (chloroform or DMSO) using tetramethylsilane (TMS; *δ* = 0) as the internal reference. UV–vis absorption spectra were measured on a Shimadzu UV‐2600 spectrophotometer. Photoluminescence (PL) spectra were recorded on a Perkin‐Elmer LS 55 spectrofluorometer. Mass spectra were recorded on a Thermo Scientific™ Dionex UltiMate 3000 LC/Exactive™ Plus Orbitrap MS. The number average (*M*_n_) and weight average (*M*_w_) molecular weights and polydispersity indices (PDIs) of the polymers were estimated by Waters Associates gel permeation chromatography (GPC 2414) system equipped with RI and UV detectors. Confocal laser scanning microscopic (CLSM) images were obtained on the confocal microscope (Zeiss Laser Scanning Confocal Microscope). The cell viability analysis for estimating cytotoxicity was collected using a microplate reader (Tecan Infinite M200PRO). Dulbecco's modified Eagle's medium (DMEM), fetal bovine serum (FBS), phosphate‐buffered saline (PBS), Streptomycin, and Penicillin were purchased from Gibco. 2′,7′‐Dichlorofluorescin diacetate (DCFH‐DA) and Chlorin e6 (Ce6) were provided by Yeasen Co. Ltd. 9, 10‐Anthracenediyl‐bis(methylene)dim alonic acid (ABDA) was provided by Sigma‐Aldrich. Propidium iodide (PI), Cell Counting Kit‐8 (CCK8), and One Step TUNEL Apoptosis Assay Kit were purchased from Beyotime Biotechnology. Mouse peripheral blood platelet isolate was purchased from Solarbio. Anti‐CD4 and CD8 antibodies (Flow cytometry) were purchased from BioLegend Co. Ltd. Anti‐P‐selectin, CD61, and CD41 antibodies (Western blot) were purchased from Biossci. CD44 antibody was purchased from Abclonal. Mouse IL‐6 and IFN‐α enzyme‐linked immunosorbent assay (ELISA) kits were purchased from Invitrogen. Mouse AMH and E_2_ kits were purchased from Immunoway. Anti‐CD8 (Immunofluorescence staining) was purchased from Biossci.

### 2.2 Synthesis and characterization

**2,2′‐((2,2‐Bis(4‐(2‐(2‐bromoethoxy)ethoxy)phenyl)ethene‐1,1‐diyl)bis(4,1‐phenylene))bis(4,4,5,5‐tetramethyl‐1,3,2‐dioxaborolane) (1)**. **1** was synthesized using methods reported in the literature.^70^ **PF3**: To a mixture of **1** (200 mg, 0.217 mmol), **2** (57.3 mg, 0.174 mmol), **3** (27.2 mg, 0.043 mmol), and Pd(PPh_3_)_4_ (12 mg, 0.01 mmol) in a 20 ml round‐bottomed flask. Toluene (5 ml) and 2 M K_2_CO_3_ solution (1 ml) were degassed by bubbling with nitrogen for 30 min and then added to the flask. The mixture was vigorously stirred at 90°C under nitrogen protection for 24 h. After cooling to room temperature, the mixture was extracted with dichloromethane. The combined organic layers were washed with water and then dried over anhydrous MgSO_4_. The polymer solution was filtered and precipitated in methanol, and then dried under vacuum for 24 h to afford the neutral polymer **PF3** as a dark red solid in 61.8% yield. ^1^H NMR (500 MHz, CDCl_3_), *δ* (TMS, ppm): 7.99 (br), 7.85 (br), 7.65 (br), 7.15 (br), 7.04 (br), 6.71 (br), 4.08 (br), 3.92–3.73 (m), 3.53–3.32 (m), 2.72 (br), 1.63 (br), 1.43–1.24 (m), 0.87 (br). *M*_w_ = 38,914 g/mol, PDI (*M*_w_/*M*_n_) =1.47.

**PF3‐PPh**_**3**_: **PF3** (30 mg) and triphenylphosphine (100 mg, 0.38 mmol) were dissolved in N,N‐dimethyl formamide (5 ml), and the mixture was stirred at 100°C for 48 h. The solvent was then removed by evaporation under pressure, and the residue was precipitated in EA. The crude product was washed with acetone and then dried under vacuum for 24 h to afford a dark red solid **PF3‐PPh**_**3**_ (35.4 mg). ^1^H NMR (500 MHz, DMSO), *δ* (TMS, ppm): 8.14 (br), 7.82–7.47 (m), 7.24 (br), 7.00 (br), 6.70 (br), 3.99 (br), 3.84–3.57 (m), 3.49 (br), 2.76–2.61 (m), 1.59 (br), 1.18 (br), 0.81 (br). ^31^P NMR (202 MHz, DMSO), *δ* (TMS, ppm): 24.87.

### 2.3 Preparation of Plt‐M@P

Briefly, blood was collected from mice via the heart and placed in tubes containing EDTA (1.5 mg/ml) and stored at 4°C. The blood sample should be subjected to platelet extraction within 24 h. Equal volumes of PBS were used to dilute the blood samples. The volume ratio of diluted blood sample to platelet isolate was 1:1. Immediately afterward, the samples were centrifuged with a centrifugal force of 200*g* and a centrifugation time of 15 min. Note that the brakes cannot be applied after the sample centrifugation is completed. Carefully aspirate the platelet‐rich plasma (top layer) from the sample and transfer to a new centrifuge tube. PBS was added to the plasma for washing, and after washing, it was centrifuged again (500*g*, 20 min). Platelets are obtained after two repeated washes. Platelet membranes are obtained by repeated freeze‐thawing of platelets.^71^ Briefly, platelets are placed in liquid nitrogen and frozen, then placed at room temperature to dissolve, and the operation is repeated five times. Platelet membranes were then obtained by high‐speed centrifugation (4000*g*, 15 min).

The preparation of Plt‐M@P is referred to the previous studies.^72^, ^73^ Briefly, to 2.0 mg PF3‐PPh_3_ dissolved in 0.4 ml of DMSO. Subsequently, to 4.0 mg PLGA dissolved in 2.0 ml of acetonitrile, PF3‐PPh_3_ was added dropwise under 3 min sonication (power, 100 W) to form the PLGA/PF3‐PPh_3_. Next, 4.0 mg Platelet membrane (Plt‐M) was dissolved in 1.0 ml deionized water under ultrasound (power, 100 W). The PF3‐PPh_3_ and Plt‐M were coextruded through a polycarbonate membrane (100 nm). Organic solvents were then removed through a centrifugal filter and the NPs were diluted in DEPC‐treated water.

### 2.4 Density functional theory and time‐dependent density functional theory calculations

Density functional theory (DFT) and time‐dependent density functional theory (TD‐DFT) calculations were calculated using the Gaussian 09 program package. The state geometries were optimized by DFT at B3LYP/6‐31G* method, without imposing any symmetry constraints. The computations of the singlet and triplet transition energy levels were carried out by TD‐DFT on B3LYP/6‐311G** method based on the optimized ground state geometry, which have reasonably predicted the singlet‐triplet energy gap (ΔE_ST_).

### 2.5 Fluorescence measurement

The fluorescence of PF3‐PPh_3_, Plt‐M, PLGA/PF3‐PPh_3_, and Plt‐M@P were measured by a PerkinElmer LS 55 spectrofluorometer.

### 2.6 Dynamic light scattering

The hydrodynamic size distribution of the PF3‐PPh_3_, Plt‐M, PLGA/PF3‐PPh_3_, and Plt‐M@P were detected by Nano‐ZS ZEN3690 (Malvern Instruments) at 25°C.

### 2.7 Transmission electron microscope

The PF3‐PPh_3_, Plt‐M, PLGA/PF3‐PPh_3_, and Plt‐M@P (10 μl) were dropped on a copper mesh. Then, the phosphotungstic acid solution (10 μl) was dropped on this copper mesh to stain the samples for 3 min. After washing the copper mesh with deionized water, most of the phosphotungstic acid solution was removed. The morphologies of the Plt‐M@P NPs were observed by transmission electron microscope (TEM) (FEI Tecnai G2 20).

### 2.8 Detection of ROS in solution

The equipment involved in the photodynamic system is shown in Figure S6. Light source, is a xenon lamp, purchased from Beijing zhonghuitingcheng Technology Co. Before conducting the photosensitivity test, the light source is fixed, and the light intensity close to the table is measured to a preset intensity (100 mW cm^−2^). Since the light intensity is checked before each test is conducted, it is possible to ensure the consistency of the light intensity. 9, 10‐Anthracenediyl‐bis (methylene)‐dimalonic acid (ABDA) is used as an indicator of ROS. A solution sample was prepared by mixing 50 μM of ABDA with the photosensitizers (P3‐PPh3, PF3‐PPh3, Ce6, and Plt‐M@P) to be tested. The solution sample was added to a 3.5 cm Petri dish so that it could be well irradiated by white light. The samples were transferred to a cuvette for UV spectroscopy after a fixed time of light irradiation (30 s). It should be protected from light when performing UV spectroscopic measurements. After the samples were tested, they were added to the petri dish again, the light was started, and so on in a cycle.

### 2.9 Drug loading efficiency

For the preparation of Plt‐M@P NPs, we put 2.0 mg of PF3‐PPh_3_, and the Plt‐M@P obtained was separated by ultrafiltration centrifugation, and the mass of PF3‐PPh_3_ in it was determined by UV spectroscopy to be 1.87 mg. Therefore, the loading efficiency of the drug can be deduced to be 93.5%.

### 2.10 Cell culture

The 4T1 (Mouse breast cancer cell line) and HLF (Mouse lung fibroblasts) were purchased from the American Type Culture Collection. The 4T1 cells were maintained in RPMI‐1640 medium supplemented with 10% FBS, 100 U/ml penicillin, and streptomycin. The HLF cells were maintained in DMEM medium supplemented with 10% FBS, 100 U/ml penicillin, and streptomycin. All cells were incubated at 37°C with 5% CO_2_. The third and fourth passages were used for experiments.

### 2.11 Blocking test

Briefly, 4T1 tumor cells were coincubated with CD44 antibody (20 ng/μl) for 12 h before incubation with Plt‐M@P, and the intracellular fluorescence intensity was detected by CLSM after 24 h.

### 2.12 CCK‐8 assay

The cytotoxicity effects of P3‐PPh_3_, PF3‐PPh_3_, and Plt‐M@P on 4T1/HLF cells were tested using a CCK‐8 assay. Briefly, 4T1/HLF cells were seeded at a density of 5 × 10^3^ per well in 96‐well plates and incubated with medium containing various concentrations of NPs for 24 h. The supernatants were then discarded and replaced by 100 μl fresh medium containing 10 μl of WST‐8 reagent. After incubation for another 1 h at 37°C, absorbance was detected with a spectrophotometer (Molecular Devices) at 450 nm for viability calculation.

### 2.13 Detection of intracellular ROS

4T1 cells were incubated with Plt‐M@P NPs (PF3‐PPh_3_ 10 μg/ml) for 2 h. Then, cells were washed with PBS three times and cultured with fresh medium containing 20 μM DCFH‐DA. After incubation for 30 min, the cells in Light and Plt‐M@P + Light groups were washed with PBS and irradiated by white light (100 mW cm^−2^, 3 min). Then, the fluorescence images of the cells were taken by CLMS (Zeiss).

### 2.14 PI staining

The 4T1 cells treated by Plt‐M@P (with or without light) were washed twice with PBS. Then add 20 μl PI (50 μg/ml) to 1 ml cell culture medium, followed by gently mixing and 15‐min incubation at 37°C in the dark. Finally, the cells were visualized under the CLSM (Zeiss).

### 2.15 Western blot

The proteins of PLGA/PF3‐PPh_3_, Plt‐M@P, and platelet membrane were extracted using RIPA buffer and then denatured at 100°C for 10 min. Samples (10 μl) were separated by 10% SDS‐PAGE and then transferred to PVDF membranes. The membranes were blocked with 5% skimmed milk for 1 h at 37°C. Then, the membranes were incubated with the primary antibodies CD44 (1:1000 dilution), P‐selectin (1:500 dilution), CD61 (1:1000 dilution), and CD41 (1:1000 dilution) overnight at 4°C. Next morning, the membranes were rewarmed at room temperature for 1 h and washed in TBST three times. HRP‐conjugated secondary antibody was added, and the membranes were incubated at 37°C for 1 h. The signals were detected by the enhanced ECL system with western blot exposure.

### 2.16 Animals

BALB/c mice were purchased from Beijing HFK Bioscience. Seven‐week‐old female animals were used throughout all experiments. The animals were maintained under controlled ambient temperature (25 ± 1°C) and humidity (55 ± 5%) in artificial lighting under a 12‐h light/12‐h dark cycle. We performed all mouse studies in the context of the animal protocol approved by the Institutional Animal Care and Ethics Committee at Tongji Hospital, Tongji Medical College of Huazhong University of science and technology. To construct the animal models with small‐burden tumors, 4T1 (5 × 10^6^) cells were injected into the right back of each mouse. The sizes of subcutaneous tumors were measured every 2 days. The tumor volume was calculated as follows: volume = (length × width^2^) × 0.5.

### 2.17 Blood pharmacokinetics

Healthy 8‐week‐old mice were injected with 100 μl of Plt‐M@P (5.0 mg/ml) via tail vein. Subsequently, blood samples were taken at different time points (0.1, 1, 2, 5, 10, 24, and 36 h) after NPs injection. Quantification of Plt‐M@P content in blood samples using the In Vivo Imaging System (IVIS) imaging system.

### 2.18 In vivo metabolic analysis of Plt‐M@P

Healthy 8‐week‐old mice were injected with 100 μl of Plt‐M@P (5.0 mg/ml) via tail vein. NPs accumulation in the heart, liver, spleen, lungs, and kidneys was measured and quantified by the IVIS imaging system at 24, 36, 48, and 60 h post‐injection.

### 2.19 Real‐time imaging in vivo

When the volumes of tumors reached around 200 mm^3^, 100 μl of Plt‐M@P (5.0 mg/ml) was injected via the tail vein. Subsequently, the fluorescence distribution in the mice was examined using the IVIS imaging system at 0.5, 1, 2, 3, 5, 8, 12, and 24 h of NPs injection.

### 2.20 Anti‐tumor therapy in vivo

When the volumes of tumors reached around 40 mm^3^, tumor‐bearing mice were randomly divided into four groups. Mice were injected with 100 μl PBS or Plt‐M@P (5.0 mg/ml). After 24 h of injection, the tumor areas of Light and Plt‐M@P (+Light) groups were exposed to white light (200 mW cm^−2^) for 20 min. The treatment was repeated every 3 days for 15 days. Tumor sizes were measured every 2 days. Then the mice were sacrificed for evaluation.

In another animal model with large tumor burdens, 5 × 10^6^ 4T1 cells were subcutaneously injected into the right back of BALB/c mice. When the volumes of tumors reached around 200 mm^3^, tumor‐bearing mice were randomly divided into four groups (PBS, PDT, anti‐PD‐L1, and PDT+ anti‐PD‐L1). In the PDT and PDT+ anti‐PD‐L1 groups, 200 μl of Plt‐M@P (5.0 mg/ml) were intravenously injected every 3 days with a total of four times. After 24 h of NPs injection, the tumors were exposed to white light (200 mW cm^−2^). In the PDT+ anti‐PD‐L1 group, the irradiated mice were then intravenously injected with 40 μg anti‐PD‐L1/mouse. At Day 13, the mice were sacrificed for evaluation.

### 2.21 Detection of the bio‐distribution of NPs

To detect the bio‐distribution of PLGA/P3‐PPh_3_ and Plt‐M@P in vivo, BALB/c mice with large subcutaneous tumors were used. 4T1 (5 × 10^6^) cells were injected into the right back of each mouse. When the volumes of tumors reached around 200 mm^3^, the mice were injected with 100 μl PLGA/P3‐PPh_3_ (5.0 mg/ml) or Plt‐M@P (5.0 mg/ml). After 24 h, the mice were euthanized and dissected, the tumor, spleen, liver, lung, heart, and kidney were obtained. Then the fluorescence signal in the organs was detected by the IVIS Spectrum imaging system (Ex = 500 nm, Em = 680 nm). Immediately afterward, the tissues were frozen sectioned and the accumulation of NPs in the tissues was observed by CLSM.

### 2.22 TUNEL staining

The apoptotic of tumor cells was detected by using the One Step TUNEL Apoptosis Assay Kit. According to the manufacturer's instructions, the paraffin section tissue was incubated with terminal deoxynucleotidyl transferase‐mediated nick‐end labeling (TUNEL) reaction mixture at 37°C for 1 h. Then the nuclei were stained with DAPI. Green fluorescein‐labeled apoptotic cells were examined with CLSM (Zeiss).

### 2.23 Flow cytometry

To detect the anti‐tumor immune effects induced by PDT and anti‐PD‐L1, the peripheral blood of each group of mice was collected into ETDA‐coated tubes. Then, 1 × 10^5^ cells were labeled with anti‐CD4/FITC and CD8/APC antibodies for 20 min and washed two times with PBS. The cells were resuspended in 100 μl FACS buffer and then analyzed by flow cytometry (Beckman, CytoFLEX S).

### 2.24 Immunofluorescence

Histological sections (4 μm thick) of organs and tumors were prepared. Deparaffinization was performed after the slides were heated at 65°C for 2 h. Then rehydration was performed in graded alcohols (100%, 95%, 85%, and 75%). Subsequently, the sections were washed by PBS three times. Then, the sections received antigen retrieval by sodium citrate and blocked with 10% normal goat serum for 30 min at 37°C, followed by incubated with anti‐CD8/CD44 antibody at 4°C overnight. The next morning, the sections were incubated with Alexa Fluor 488‐labeled Goat Anti‐Rabbit IgG (1:200 dilution) or FITC‐labeled Goat Anti‐Rabbit IgG (1:200 dilution) for 1 h at 37°C. Then, images were obtained through the CLSM.

### 2.25 Cytokine detection

The plasma levels of IL6 and IFN‐α were measured using ELISA kits. Plasma samples were extracted from the mice and diluted for analysis according to the ELISA Development Guide. All measurements were carried out in triplicate. Optical density (OD) was measured at a wavelength of 450 nm with a spectrophotometer (Molecular Devices).

### 2.26 Hematoxylin and eosin staining

Histological sections (4 μm thick) of the tissues were heated at 65°C for 2 h, followed by deparaffinization. Then, the process of rehydration was performed in graded alcohols (100%, 95%, 85%, and 75%). Next, sections were stained with hematoxylin and eosin (H&E). Images were obtained through the microscope (Olympus).

### 2.27 Ovarian follicle counting

Paraffin‐embedded ovaries were sectioned to obtain 5‐mm thick serial sections, and one of the four continuous ovarian sections was chosen to count the follicles. Follicles at different stages, including primordial follicles, primary follicles, secondary follicles, antral follicles, and atretic follicles, were determined as previously described.^74^ Follicles surrounded by squamous granulosa cells were identified as primordial follicles. Primary follicles have a single layer of cuboidal granulosa cells. Secondary follicles possessed more than one layer of granulosa cells without antrum. Antral follicles have one or two antra. Atretic follicles lose the normal morphology and show nuclear lysis, nuclear pyknosis, and nuclear fragmentation.

### 2.28 Testing of serum levels of estradiol and AMH by ELISA

The serum of each group was used to measure the concentrations of estradiol (E2) and AMH by using ELISA Kit. The experiments were conducted according to the instructions. All measurements were carried out in triplicate. OD was measured at a wavelength of 450 nm with a spectrophotometer (Molecular Devices).

### 2.29 Statistical analysis

Data were presented as mean ± standard deviation (SD). Statistical analysis was performed by GraphPad Prism (version 8.1.2; GraphPad Software). *p* values <0.05*, <0.01** and <0.001*** were considered to be statistically significant.

## 3 RESULTS

### 3.1 Synthesis and characterization of PF3‐PPh_3_ and Plt‐M@P

**FIGURE 2:**
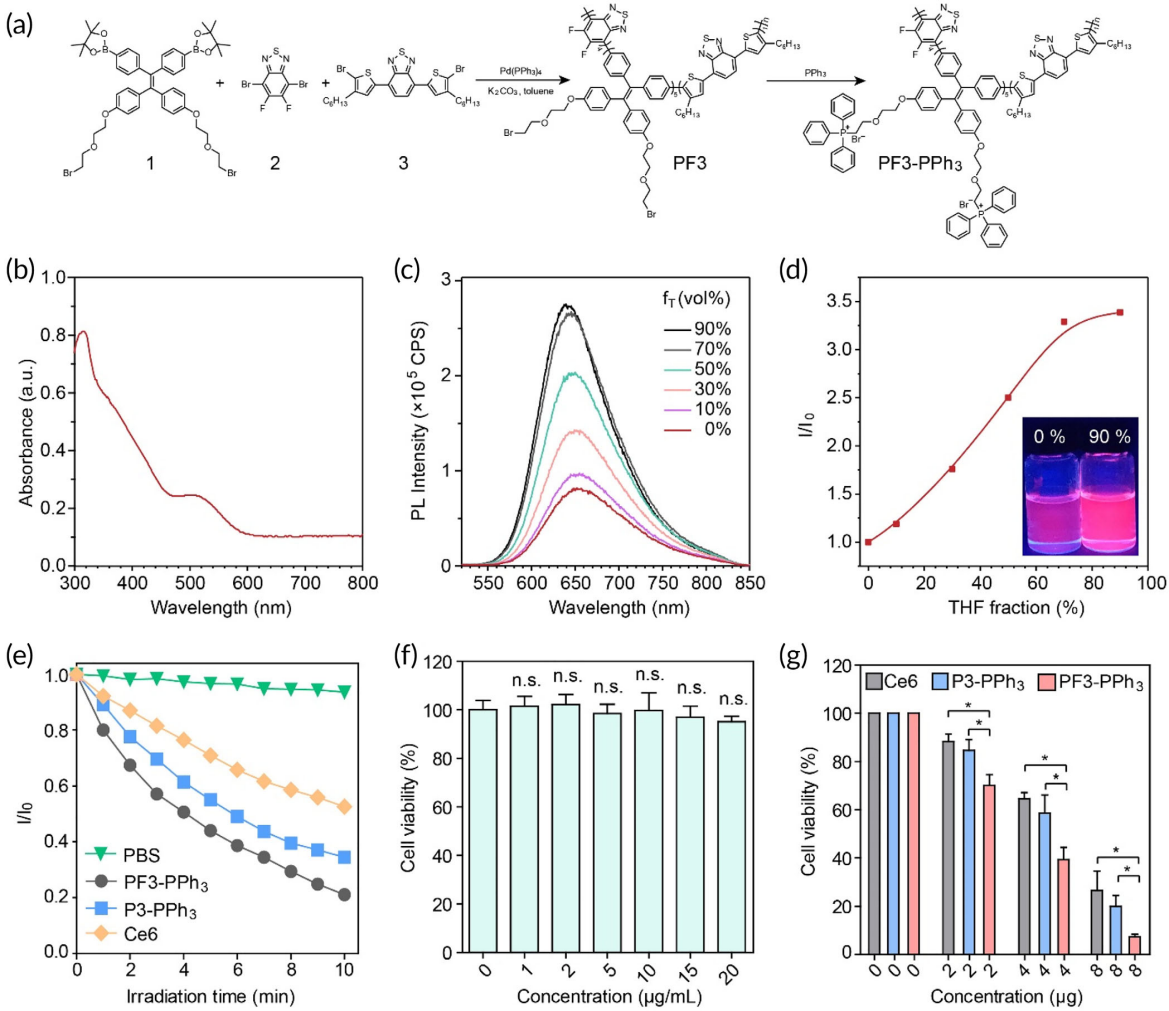
Synthesis and characterization of PF3‐PPh_3_. (a) Synthetic route of PF3‐PPh_3_. (b) UV–Vis absorption spectrum of PF3‐PPh_3_ in DMSO solution. (c) PL spectra of PF3‐PPh_3_ in DMSO/THF mixtures with different THF fraction (*f*_T_). Ex = 500 nm. (d) Plot of *I*/*I*_0_ of PF3‐PPh_3_ versus the compositions of the solution mixtures. *I*_0_ = PL intensity in pure DMSO solution. (e) The relative production of ROS under white light irradiation (10 mW cm^−2^) was measured using ABDA (50 μM) for Ce6 (10 μg/ml), P3‐PPh_3_ (10 μg/ml), and PF3‐PPh_3_ (10 μg/ml). ABDA is an indicator of ROS, which is decomposed in the presence of ROS, allowing its quantification by UV spectroscopy. I = UV absorption value of ABDA before light irradiation. *I*_0_ = UV absorption value of ABDA after light irradiation. (f) Cell viability of 4T1 cells after incubation with different concentrations of PF3‐PPh_3_ for 24 h. (g) Cell viability of 4T1 cells incubated with different concentrations of Ce6, P3‐PPh_3_, or PF3‐PPh_3_ for 24 h and treated with white light (100 mW cm^−2^, 5 min). Data were reported as mean ± SD and analyzed by two‐sided Student's *t*‐test. *p < 0.05, n.s., not significant; ROS, reactive oxygen species.

Previously, we have successfully constructed polyelectrolyte P3‐PPh_3_ with AIE properties.^70^ To further enhance the performance of this AIE photosensitizer system, PF3‐PPh_3_ was designed and successfully synthesized, and its synthesis path is shown in Figure 2a. Compound 1 was prepared using a previously reported method.^70^ The neutral polymer PF3 was synthesized by using Suzuki polycondensation of the monomers compounds 1, 2, and 3 with feed molar ratios of 5:4:1 in moderate yield of 61.8%. PF3 was then reacted with triphenylphosphine to yield the final product. The detailed synthetic methods and NMR characterization data were given in the experimental section (Figures S1–S3). The expected molecular structure of PF3 and PF3‐PPh_3_ was confirmed from the NMR spectroscopic analyses. For example, peak at *δ* = 24.87 ppm in the ^31^P NMR spectrum of PF3‐PPh_3_ originated from the phosphorus on its sidechains. The weight‐average molecular weight of PF3 was 38,914 g/mol and the polydispersity was 1.47, determined by gel permeation chromatography using chloroform as the solvent and polystyrene as the standard. The ultraviolet–visible (UV–vis) absorption spectra of PF3‐PPh_3_ in DMSO solution are shown in Figure 2b, where PF3‐PPh_3_ was completely dissolved in DMSO. The broad absorption spectrum from 350 to 600 nm was attributed to the intramolecular charge transfer on the donor‐acceptor main backbone structure. The PL spectra of PF3‐PPh_3_ in dilute DMSO solution exhibited broad and moderate peaks due to the random‐coil configuration of the polymer, which partly restricted the intramolecular rotations and blocked the nonradiative decay channel.^75^ When THF as a poor solvent for PF3‐PPh_3_ was gradually added to the DMSO solution, the PL intensity of PF3‐PPh_3_ was obviously enhanced, displaying typical AIE enhancement (AIEE) feature (Figure 2c). The red emission was located at about 650 nm in the 9:1 vol% THF/DMSO mixture and the intensity was 3.3‐fold higher than the isolated species in pure DMSO solution (Figure 2d). To verify that PF3‐PPh_3_ could promote the generation of singlet‐oxygen as a photosensitizer to achieve effective PDT, 9, 10‐Anthracenediyl‐bis (methylene)‐dimalonic acid (ABDA) was used in the experiment (Figure 2e). In the presence of PF3‐Ph3, the UV absorption value of ABDA decreased rapidly under light irradiation, while ABDA alone decreased very little under 10 min of irradiation, implying that PF3‐Ph3 has good photosensitizing ability. More importantly, PF3‐PPh_3_ is a stronger photosensitizer compared to our previously reported P3‐PPh_3_, exemplified by a higher level of ROS production under the same light irradiation power and time (Figure 2e). Also, the commercial photosensitizer Ce6 has a poorer ROS yield than PF3‐PPh_3_ under white light irradiation. To study the effect of additional two fluorine substituents on molecular configuration and the frontier molecular orbitals of polymers, DFT calculations on the conjugated fragments M3 of P3‐PPh_3_ and MF3 of PF3‐PPh_3_ were carried out. The long alkyl chains are replaced by the methyl groups to simplify the calculations. Compared with M3, MF3 obtains a smaller singlet‐triplet energy gap (ΔE_ST_) (Figure S4). Such changes can facilitate the conversion of singlet excitons to triplet excitons by an intersystem crossing process and populate more triplet excited species of the photosensitizers for singlet oxygen generation. The cells viability was greater than 95% even when 4 T1 cells (a mouse breast cancer cell) were incubated with 20 μg/ml PF3‐PPh_3_ for 24 h in the dark, demonstrating the low cytotoxicity of PF3‐PPh_3_ (Figure 2f). However, after 5 min of white light irradiation (100 mW cm^−2^), the number of dead cells increased rapidly due to the ROS generated by PF3‐PPh_3_. The dose‐dependent cytotoxicity results showed that the cell viability of PF3‐PPh_3_ decreased approximately significantly with increasing concentration, with cell viability below 10% at 8 μg/ml (Figure 2g). Under the same photosensitizer concentration and light conditions, the cell killing effect of PF3‐PPh_3_ was stronger than that of P3‐PPh_3_ and Ce6, indicating a better photodynamic effect of PF3‐PPh_3_ (Figure 2g). Altogether, these results indicated the new AIE photosensitizer, PF3‐PPh_3_, possesses an improved performance, including excellent photophysical and photodynamic properties.

**FIGURE 3:**
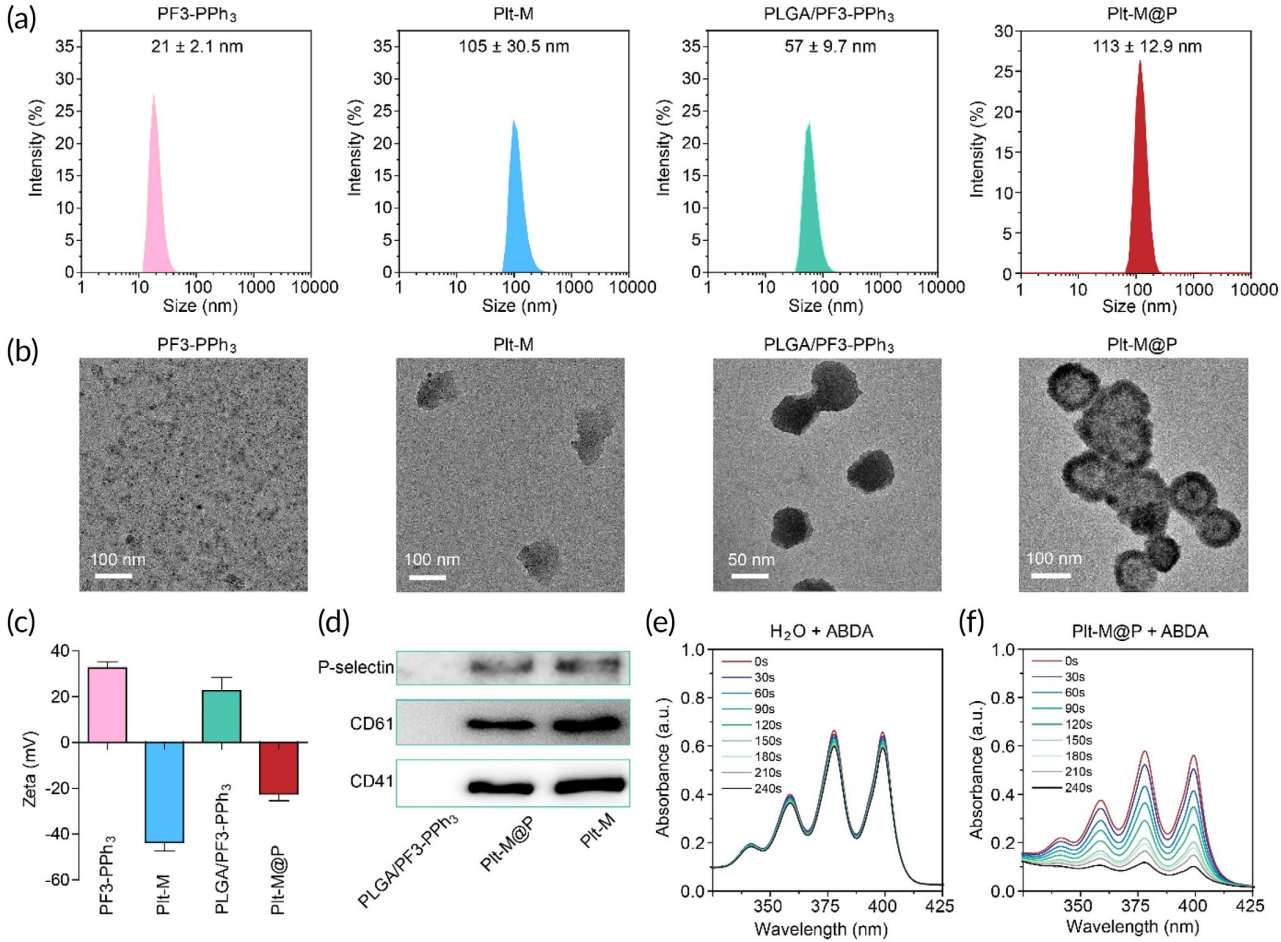
Characterization of Plt‐M@P. (a) DLS to detect the particle size of PF3‐PPh_3_, Plt‐M, PLGA/PF3‐PPh_3_, and Plt‐M@P. (b) The particle size and morphology of PF3‐PPh_3_, Plt‐M, PLGA/PF3‐PPh_3_, and Plt‐M@P were examined by TEM. (c) The zeta potentials of PF3‐PPh_3_, Plt‐M, PLGA/PF3‐PPh_3_, and Plt‐M@P. (d) Protein profiling analysis of PLGA/PF3‐PPh_3_, Plt‐M@P, and Plt‐M. (e, f) ROS production in (e) H_2_O and (f) Plt‐M@P was detected using ABDA under white light irradiation. The concentration of ABDA is 50 μM. The concentration of PF3‐PPh_3_ is 10 μg/ml. The white light intensity is 100 mW cm^−2^. DLS, dynamic light scattering; TEM, transmission electron microscopy.

Encouraged by the in vitro performance of PF3‐PPh_3_, we further explored the in vivo application by using the platelet membrane‐camouflaged strategy to prepare Plt‐M@P NPs. The UV–Vis absorption and PL spectra of Plt‐M@P were shown in Figure S5a and Figure S5b, respectively. The NP sizes of PF3‐PPh3, Plt‐M, PLGA/PF3‐PPh3 and Plt‐M@P NPs are 21 ± 2.1, 105 ± 30.5, 57 ± 9.7, and 113 ± 12.9 nm, respectively, as shown in Figure 3a. The particle size and morphology of the NPs were then observed by transmission electron microscopy (TEM) and displayed in Figure 3b. TEM results showed that the platelet membranes (Plt‐M) were irregularly lamellar. PLGA/PF3‐PPh_3_ was irregularly spherical with a diameter of about 100 nm. Plt‐M@P has a particle size of about 120 nm, and its outermost layer has a platelet membrane of about 10 nm thickness. The zeta potentials of these NPs were then determined to be 32.5, −43.5, 22.6, and −22.5 mV for PF3‐PPh_3_, Plt‐M, PLGA/PF3‐PPh_3_, and Plt‐M@P, respectively (Figure 3c). Subsequently, we used Western blotting to confirm that platelet‐specific proteins such as P‐selectin, CD61, and CD41 were inherited by Plt‐M@P NPs and not seen on the PLGA/PF3‐PPh_3_ complex (Figure 3d),^68^ suggesting Plt‐M@P indeed contains platelet membranes. Finally, we tested the photosensitizing ability of Plt‐M@P using 9, 10‐Anthracenediyl‐bis (methylene) dimalonic acid (ABDA) as an indicator of ROS which is rapidly decomposed in the presence of ROS.^76^ In the absence of Plt‐M@P, ABDA was not decomposed upon light irradiation, suggesting its UV–Vis absorption value did not change if just under light irradiation (Figure 3e). On the other hand, when Plt‐M@P was present in the solution, the UV–Vis absorption value of ABDA decreased rapidly, confirming the ROS production in the presence of Plt‐M@P (Figure 3f and Figure S7). Finally, we calculated a drug loading efficiency of 93.5% for Plt‐M@P. In conclusion, we have demonstrated the platelet membrane‐camouflaged AIE NPs also possess excellent fluorescence properties and photosensitizing capability in vitro.

### 3.2 Plt‐M@P‐mediated PDT drives tumor cell apoptosis

**FIGURE 4:**
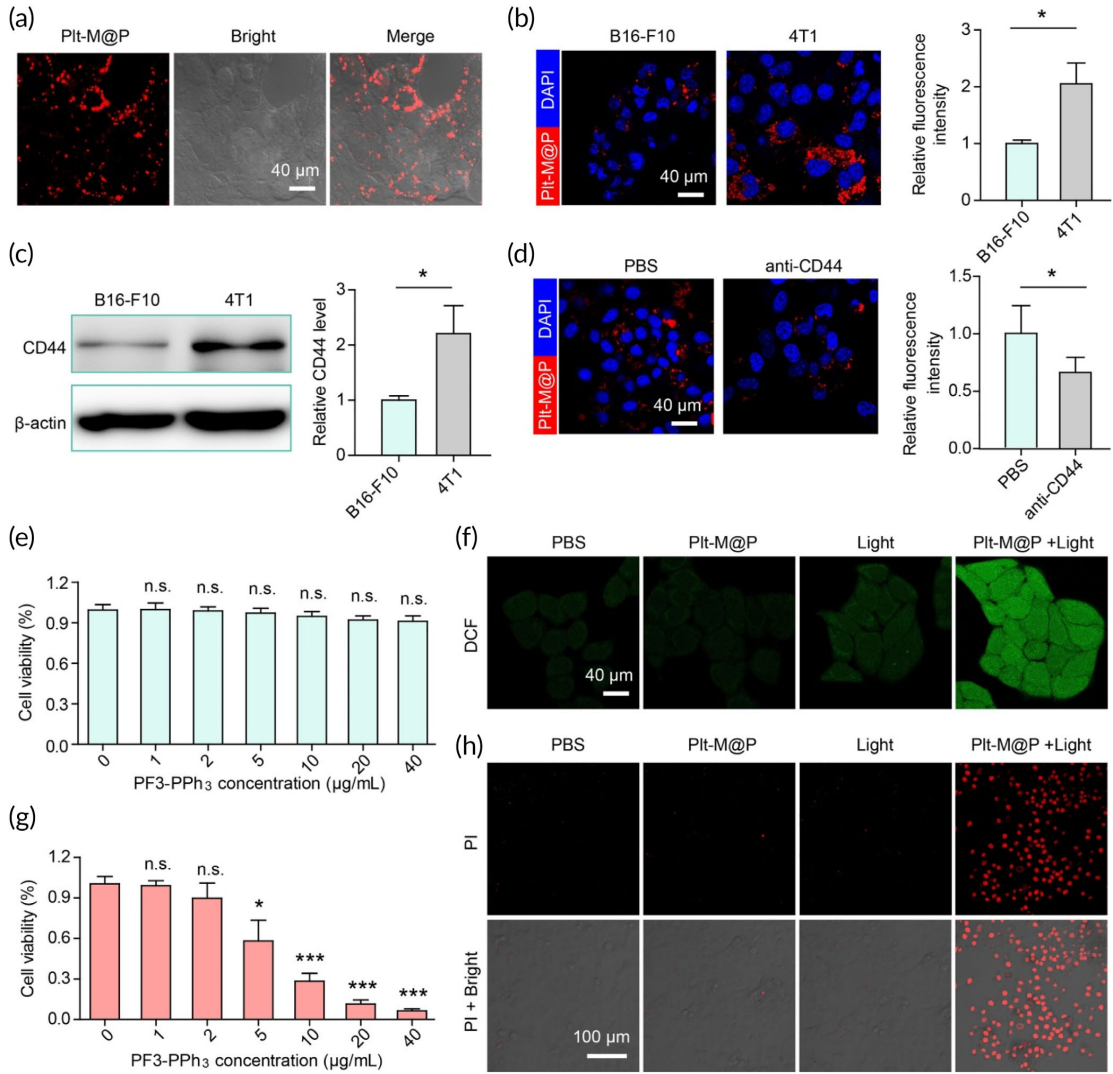
Plt‐M@P‐mediated PDT drives tumor cell apoptosis. (a) 4T1 cells were incubated with Plt‐M@P for 24 h, and then the fluorescence signals were detected by CLSM. The content of PF3‐PPh_3_ was 10 μg/ml. Ex = 488 nm, Em = 660–700 nm. Scale bar = 40 μm. (b) Uptake of Plt‐M@P by B16‐F10 and 4T1 cells for the same incubation concentration and time. (c) CD44 expression levels in B16‐F10 and 4T1 cells. (d) Changes in the uptake of Plt‐M@P after incubation of 4T1 cells with CD44 antibody. (e) Viability of 4T1 cells treated with different concentration of Plt‐M@P without light irradiation. (f) The ROS levels in 4T1 cells were detected by DCFH‐DA. Light intensity: 100 mW cm^−2^; Irradiation time: 3 min. Ex = 488 nm; Em = 520–540 nm. Scale bar = 40 μm. (g) Viability of 4T1 cells treated with different concentrations of Plt‐M@P under light irradiation by CCK‐8 assay. Light intensity: 100 mW cm^−2^; Irradiation time: 3 min. (h) PI staining was performed to detect the apoptosis of 4T1 cells after PDT. The red channel is PI, and the Merge channel is the superposition of PI and bright field. Ex = 633 nm; Em = 680 nm. Scale bar = 100 μm. Data were reported as mean ± SD and analyzed by two‐sided Student's t‐test. *p < 0.05，***p < 0.001. CLSM, confocal laser scanning microscopy; PDT, photodynamic therapy; n.s., not significant; ROS, reactive oxygen species.

First, we clarified that Plt‐M@P can be taken up by tumor cells. After the Plt‐M@P were incubated with mouse breast cancer cells (4T1) for 24 h, strong red fluorescence can be observed in the cells by confocal laser scanning microscopy (CLSM), indicating successful cell uptake of the Plt‐M@P (Figure 4a). Comparing 4T1 cells with B16‐F10 cells, the CLSM results suggested that 4T1 can take up more Plt‐M@P (Figure 4b). This can be explained by the different CD44 expression levels between 4T1 and B16‐F10 cells, which were found to be higher in 4T1 than B16‐F10 cells (Figure 4c). Previous studies suggested platelet camouflaged NPs may specifically bind CD44 on cancer cells via P‐selectin, thus enabling active tumor targeting of NPs.^66^, ^77^ To verify this hypothesis, we blocked CD44 on tumor cells by using CD44 antibody and then incubated Plt‐M@P. CLSM revealed a decrease in the NPs uptake upon CD44 blockage (Figure 4d). This result suggested that CD44 may be one of the pathways through which Plt‐M@P enters cells, at least partially influencing the uptake of NPs by 4T1 cells. Then, CCK‐8 assay was employed to test the cytotoxicity of Plt‐M@P. With the concentration of PF3‐PPh_3_ increasing from 0 to 40 μg/ml, cell activity was not affected (Figure 4e). In addition, Plt‐M@P did not show significant cytotoxicity in lung fibroblast HLF, suggesting that Plt‐M@P has good biocompatibility (Figure S8). To investigate the photosensitizing properties of Plt‐M@P in cells, we used a ROS indicator, 2′, 7′‐dichlorodihydrofluorescein diacetate (DCFH‐DA) to detect intracellular ROS. After being co‐incubated with DCFH‐DA for 20 min, the 4T1 cells were irradiated with white light (100 mW cm^−2^) for 3 min and a strong green fluorescence was observed in Plt‐M@P treated cells, indicating that Plt‐M@P produce excessive ROS under light irradiation (Figure 4f and S9). Excess ROS in the cells contributes to the oxidation of intracellular biomolecules, which triggers cell death.^78^, ^79^ The viability of 4T1 treated with different concentrations of Plt‐M@P under light conditions was first measured by the CCK8 method, as shown in Figure 4g. The results showed that the viability of 4T1 cells gradually decreased with increasing concentration of Plt‐M@P, indicating that Plt‐M@P has a photodynamic therapeutic effect and is concentration‐dependent. The subsequent use of PI staining also confirmed that Plt‐M@P NP‐mediated PDT was effective in killing tumor cells (Figure 4h). In conclusion, Plt‐M@P can be actively targeted into cells via the P selectin‐CD44 pathway after platelet membrane camouflage and exert antitumor PDT.

### 3.3 Pharmacokinetics and tumor targeting of Plt‐M@P in vivo

**FIGURE 5:**
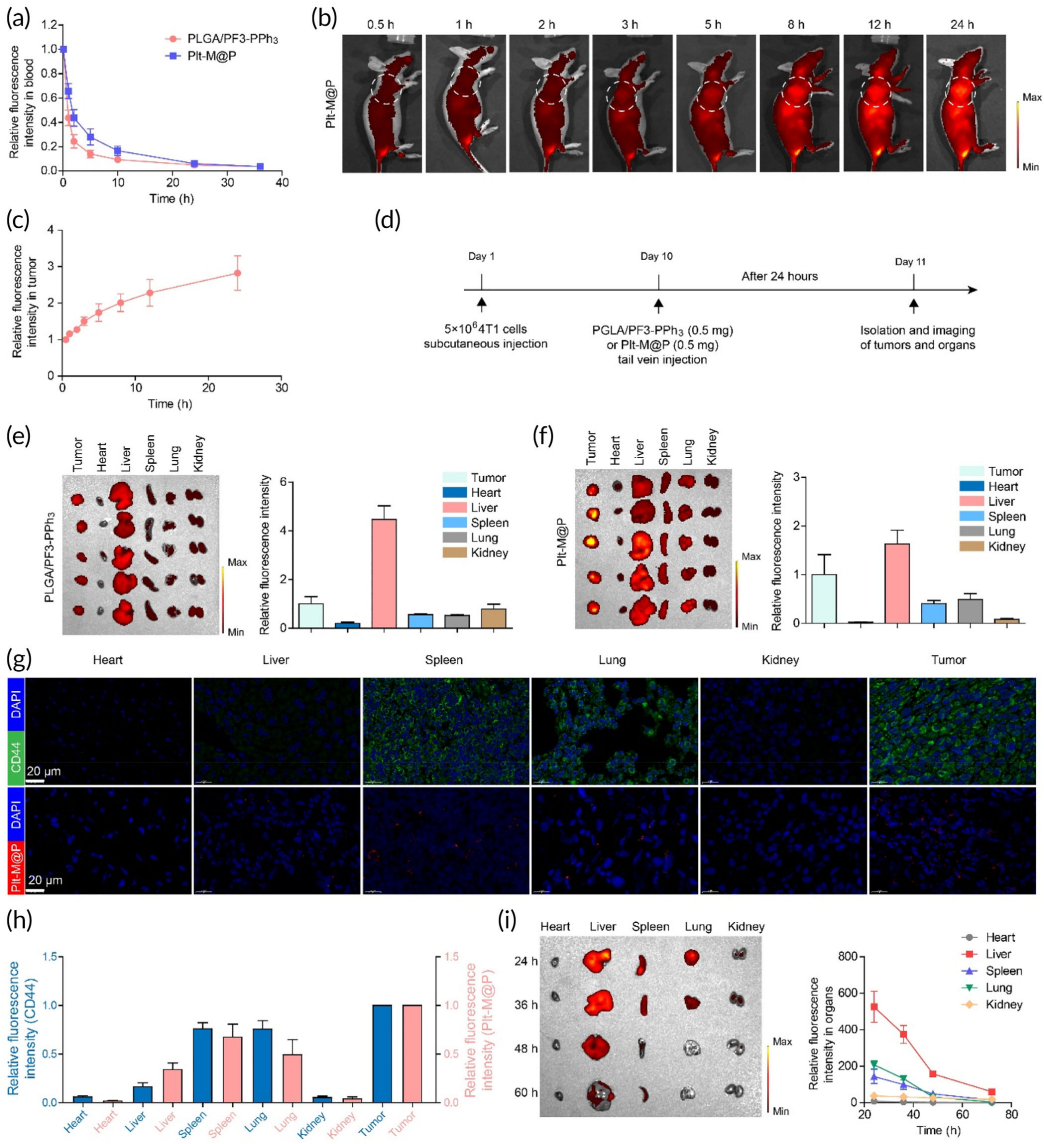
Tumor targeting of Plt‐M@P in tumor models with large burdens (>200 mm^3^). (a) Metabolic kinetics of PLGA/PF3‐PPh_3_ and Plt‐M@P in the blood of healthy mice (*n* = 3). (b) Bio‐distribution of Plt‐M@P in 4T1 tumor‐bearing mice. The white dashed circle shows the area where the tumor is located. (c) Quantitative analysis of fluorescence intensity in the tumor region. (d) Protocol for imaging the distribution of PLGA/PF3‐PPh_3_ and Plt‐M@P in tumor‐bearing mice. (e) Bio‐distribution and relative quantification (total fluorescence signal) of PLGA/PF3‐PPh_3_ in tumor, heart, liver, spleen, lung, and kidney (*n* = 5). (f) Bio‐distribution and relative quantification (total fluorescence signal) of Plt‐M@P in tumor, heart, liver, spleen, lung, and kidney (*n* = 5). (g) Expression levels of CD44 (top row) in heart, liver, spleen, lung, kidney, and tumor tissues, and the distribution of Plt‐M@P nanoparticles (bottom row) in them. (h) Relative quantitative analysis of CD44 and Plt‐M@P levels in heart, liver, spleen, lung, kidney, and tumor. (i) Metabolic pathways of Plt‐M@P in healthy mice (*n* = 3). Data were reported as mean ± SD.

The long circulation of NPs in vivo is not only the basis for the enhanced permeability and retention (EPR) effect, but also allows for better active targeting. The pharmacokinetic results show that Plt‐M@P has a longer circulation time in the body than PLGA/PF3‐PPh_3_ NPs (without platelet membrane) (Figure 5a), implying the platelet membranes can help improve the circulation. Next, to evaluate the tumor‐targeting ability of Plt‐M@P in vivo, a tumor‐bearing mouse model was established with 4T1 cells. When the tumors grew to approximately 200 mm^3^, Plt‐M@P NPs were injected into the tumor‐bearing mice via the tail vein. IVIS imaging showed that Plt‐M@P NPs gradually accumulated in the tumors, indicating their good tumor targeting properties (Figure 5b,c). Similarly, 4T1 tumor‐bearing mice were injected with PLGA/PF3‐PPh_3_ and Plt‐M@P NPs via tail vein, and IVIS was used to image their bio‐distribution in tumors and organs (Figure 5d). The bio‐distribution of the PLGA/PF3‐PPh_3_ and Plt‐M@P NPs in the tumors and organs were shown in Figure 5e and Figure 5f, respectively. Due to the presence of platelet membrane, strong fluorescence signals were observed in the tumors in the Plt‐M@P group, but much weaker in the PLGA/PF3‐PPh_3_ group. Quantitative region‐of‐interest analysis showed that the accumulation of PLGA/PF3‐PPh_3_ in the tumor was only 0.7‐fold the level of the liver, while the accumulation of Plt‐M@P in the tumor reached about 1.7‐fold the level of the liver. There are two possible reasons for this result. First, the platelet membranes of Plt‐M@P enable long circulation of the NPs, which can strengthen the EPR effect.^80^ Second, specifically expressed proteins on platelet membranes can recognize receptors on the surface of tumor cells, thus enhancing the active targeting ability of NPs, such as P‐selectin (receptor) and CD44 (ligand).^64^, ^81^, ^82^ To confirm this hypothesis, immunofluorescence staining was used to estimate the expression level of CD44 in the heart, liver, spleen, lung and kidney (Figure 5g). Among them, there was almost no CD44 expression in the heart, liver, and kidney, while a high level of expression was found in the spleen and lung. CD44 expression in the tumor tissue was also at high levels (top row of Figure 5g). Meanwhile, CLSM results confirmed the accumulation of Plt‐M@P in organs and tumors. As shown in Figure 5g (bottom row), NPs barely accumulated in the heart and kidney, but small amounts were distributed in the liver, spleen, and lung, with the largest accumulation in the tumor. The relative quantification of CD44 and Plt‐M@P levels in tissues showed a positive correlation between CD44 expression level and Plt‐M@P accumulation in all tissues and organs, except in the liver (Figure 5h). The liver has the function of detoxification, which will actively take up some foreign substances and remove them.^83^ Therefore, even if the liver does not express CD44, it will actively take up a large number of Plt‐M@P NPs and remove them. Finally, we also evaluated the metabolic pathways of Plt‐M@P in vivo (Figure 5i). The metabolic organs of Plt‐M@P include mainly the liver, lungs, and spleen, and were largely metabolized from the body by 60 h after injection. Collectively, these in vivo findings further confirmed that the NPs with platelet membrane accumulate in the tumor by passive and active targeting.

### 3.4 Assessing the efficacy of PDT in models with small tumor burdens

**FIGURE 6:**
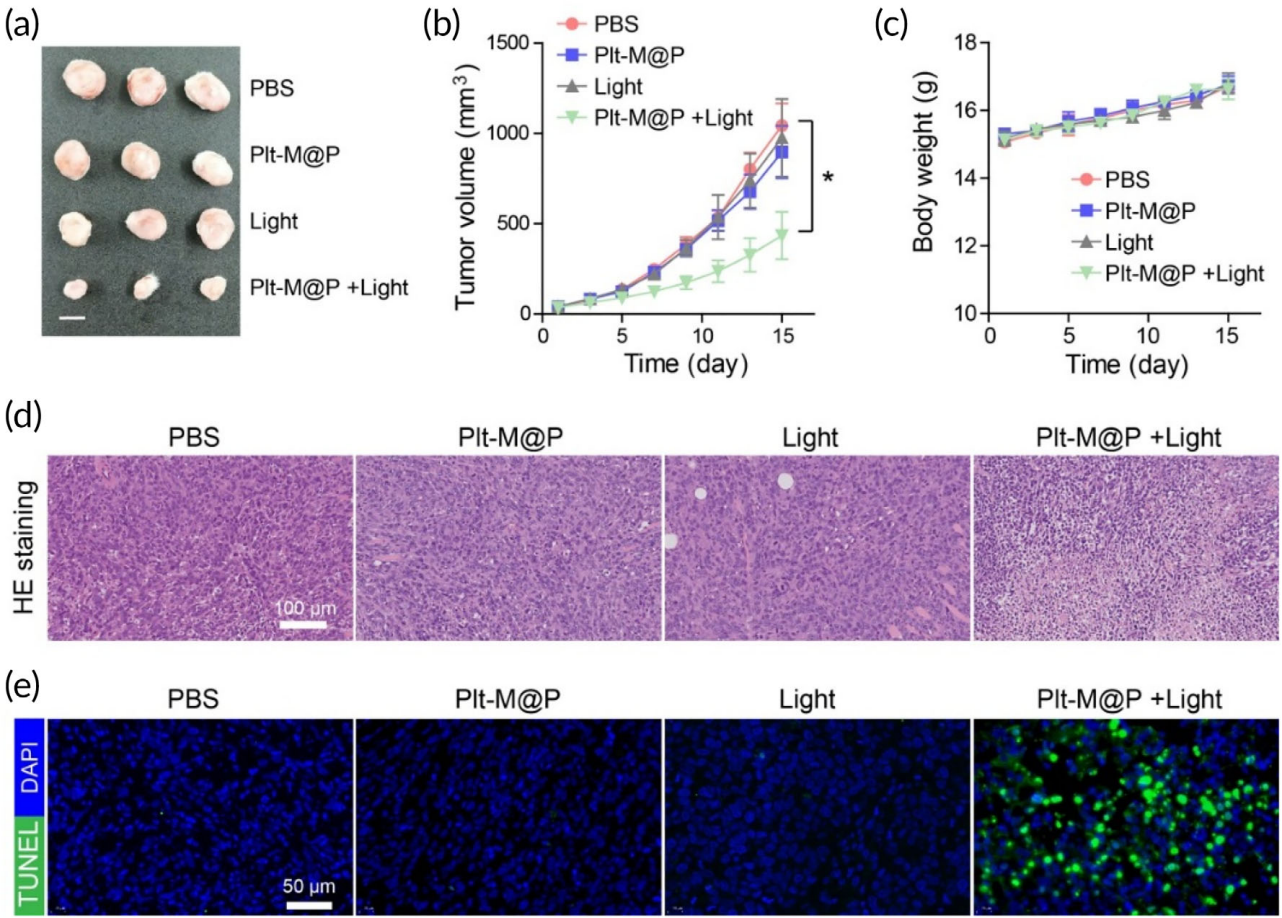
Anti‐tumor efficacy evaluation of Plt‐M@P in tumor models with small burdens (<40 mm^3^). (a) Representative tumor photographs of small load tumor models after receiving Plt‐M@P‐mediated PDT. Scale bar = 1.0 cm. (b) Tumor growth kinetics in a small‐burden tumor model after receiving Plt‐M@P‐mediated PDT. Data were reported as mean ± SD and analyzed by unpaired *t*‐test. *p < 0.05. (c) Change curves for the body weight of mice. (d) H&E staining images of tumor. Scale bar = 100 μm. (e) TUNEL staining of tumor. Scale bar = 50 μm. The blue channel is DAPI staining (Ex = 352 nm, Em = 450 nm), and the green channel is apoptotic cells (Ex = 488 nm, Em = 520 nm). Scale bar = 50 μm. PDT, photodynamic therapy; H&E, hematoxylin and eosin.

4T1 tumor‐bearing Balb/c mice were used to assess in vivo antitumor efficacy. When tumors grew to the size of ~40 mm^3^ with the small tumor burdens, PBS or Plt‐M@P were administrated intravenously into the 4T1 tumor‐bearing mice via tail vein. The growth of the tumor was significantly inhibited after the treatment with Plt‐M@P combined with light irradiation, compared with the other three groups (Figure 6a,b), which indicated the Plt‐M@P‐mediated PDT could effectively inhibit the growth of tumor with small tumor burdens. Meanwhile, the body weight of mice receiving different treatments remained stable (Figure 6c). To further explore the anti‐tumor mechanism of Plt‐M@P, we firstly examined the histopathology of tumor tissue by H&E staining. Histologic images of the tumor sections showed massive apoptosis and necrosis of tumor cells after Plt‐M@P (+Light) treatment (Figure 6d). In addition, the fluorescence images obtained using the in situ terminal deoxynucleotidyl transferase‐mediated dUTP‐biotin nick end labeling (TUNEL) staining assay presented the highest level of cell apoptosis in the tumor collected from the mice treated with Plt‐M@P (+Light) (Figure 6e). Collectively, we demonstrated that Plt‐M@P‐mediated PDT is an effective way to inhibit the growth of tumor cells in vivo.

### 3.5 PDT combined with anti‐PD‐L1 effectively suppresses large‐burden tumors

**FIGURE 7:**
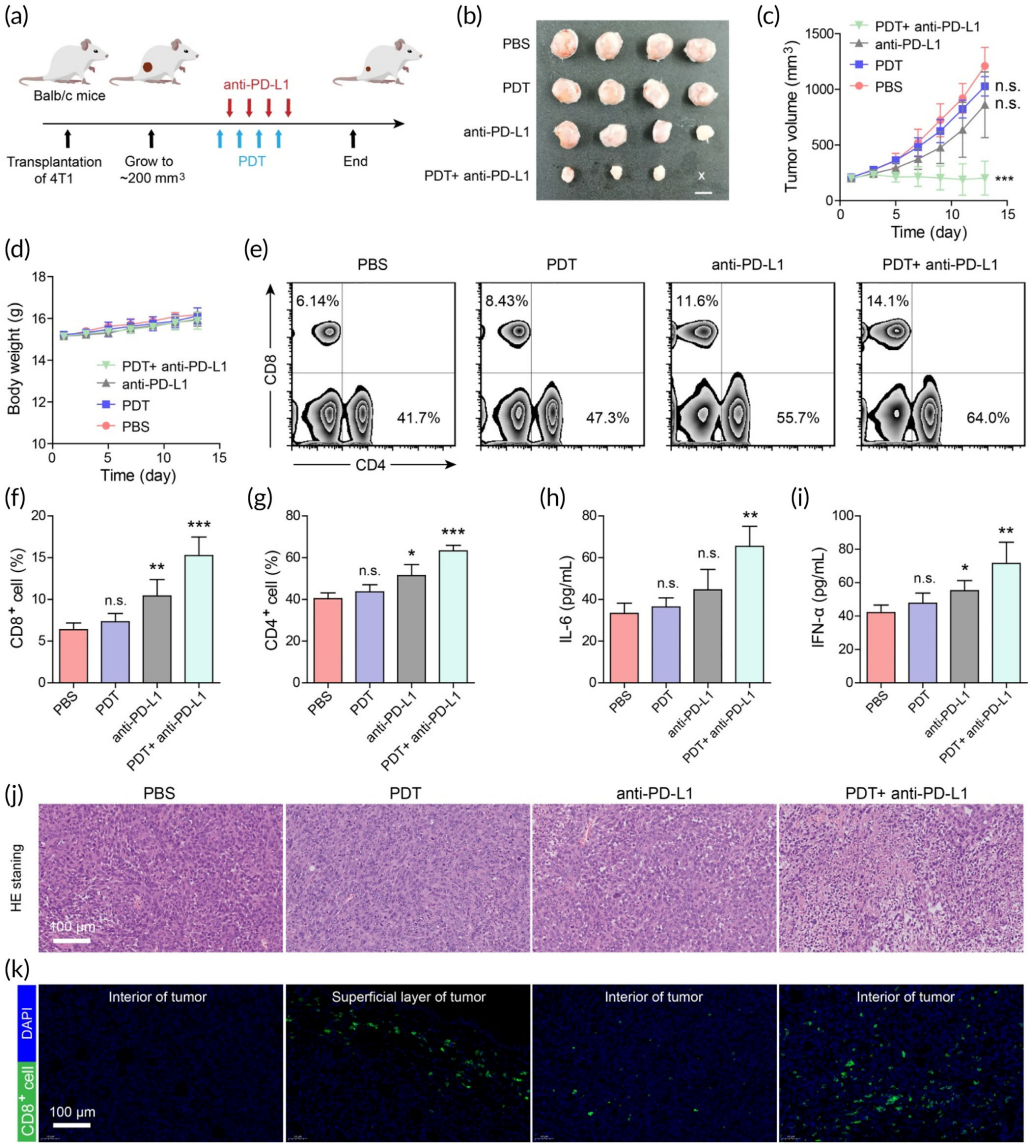
Plt‐M@P‐mediated PDT combined with anti‐PD‐L1 effectively suppresses large‐burden tumors. (a) Treatment schedule for Plt‐M@P mediated PDT and anti‐PD‐L1. (b) Photographs of excised tumors after various treatments. Scale bar = 1.0 cm. (c) Time‐dependent tumor volume growth curves after different treatments. Data were reported as mean ± SD and analyzed by unpaired *t*‐test. (d) Change curves for the body weight of mice. (e) The proportion of CD8^+^ and CD4^+^ cells in the peripheral blood of 4T1 tumor‐bearing mice after treatment. (f) Statistical analysis of the proportion of CD8^+^ cells in peripheral blood of mice after treatment. (g) Statistical analysis of the proportion of CD4^+^ cells in peripheral blood of mice after treatment. The expression levels of (h) IL‐6 and (i) IFN‐α in plasma detected by ELISA. Data were reported as mean ± SD and analyzed by two‐sided Student's *t*‐test. (j) H&E staining images of tumors. Scale bar = 100 μm. (k) CLSM images of tumor localization of CD8^+^ T cells after treatment. The blue channel is DAPI staining (Ex = 352 nm, Em = 450 nm), and the green channel is CD8 (Ex = 488 nm, Em = 520 nm). Scale bar = 100 μm. *p < 0.05, **p < 0.01，, ***p < 0.001. CLSM, confocal laser scanning microscopy; ELISA, enzyme‐linked immunosorbent assay; H&E, hematoxylin and eosin; n.s., not significant; PDT, photodynamic therapy.

Large‐burden tumors are often seen in clinical scenarios, in which patients often present with advanced tumors. We thus further explored the effect of Plt‐M@P‐mediated PDT and its combination with immune checkpoint inhibitor anti‐PD‐L1 on large‐burden tumors. 4T1 cells were injected subcutaneously to construct mouse tumor models. In the large‐burden tumor models, treatment was initiated when the tumor size reached ~200 mm^3^, which is according to the definition of large tumor in the study by Watanabe et al.^84^ The mice were randomly separated into four groups (*n* = 4) as follows: PBS, PDT, anti‐PD‐L1, and PDT+ anti‐PD‐L1. The process of tumor therapy is illustrated in Figure 7a. In PDT and PDT+ anti‐PD‐L1 groups, 100 μl of Plt‐M@P were injected via the tail vein every 3 days with a total of four times. At 24 h after the injection, mice were exposed to white light (200 mW cm^−2^) for 15 min. In the PDT+ anti‐PD‐L1 group, the mice were then intravenously administered with PD‐L1 antibody.^85^, ^86^ On Day 13 of the treatment, the mice were euthanized and the tumors were removed. Photographs of the tumors indicated that the tumors in mice treated with PDT plus anti‐PD‐L1 were smaller than those treated with other regimens (Figure 7b,c). For large‐burden tumors, combination therapy may have better antitumor effect. Watanabe's study showed that radiotherapy with anti‐PD‐1 checkpoint blockade enhanced (natural killing) NK and CD8^+^ T cell‐dependent antitumor immunity in large tumors.^84^ In addition, there was no significant change in body weight in each group, indicating an absence of severe systemic toxicity of the NPs (Figure 7d). There was no significant difference for using PDT or anti‐PD‐L1 immunotherapy alone for large tumors. For PDT, the ineffectiveness may arise from the limited tissue penetration depth of light, whereas for anti‐PD‐L1, the immunosuppressive microenvironment may be a hindrance to large tumor treatment. Therefore, we investigated why PDT plus anti‐PD‐L1 can achieve better oncologic outcomes. An important principle of tumor immunology is that tumor cells can be eliminated by T cells.^87^ The peripheral blood was collected for quantifying the fraction of CD4^+^ and CD8^+^ T cells by flow cytometry after the various treatments. The populations of CD4^+^ and CD8^+^ T cells were increased in anti‐PD‐L1 treated mice compared to untreated mice. The combination therapy with PDT and anti‐PD‐L1 showed greater increases in CD4^+^ and CD8^+^ T cell numbers than anti‐PD‐L1 alone (Figure 7e–g). In addition, cytokine released from immune cells is important in tumor immunology and suppresses tumor cell growth by a direct anti‐proliferative or pro‐apoptotic activity.^88^, ^89^ Systemic cytokines in peripheral blood were analyzed by ELISA. The results showed that IL‐6 and IFN‐α were significantly higher in mice treated with PDT plus anti‐PD‐L1 than in the other treatment groups (Figure 7h,i). It is considered that IL‐6 opposes tumor growth by mobilizing anti‐tumor T cell immune responses to attain tumor control.^90^ IFN‐α, another important pro‐inflammatory factor, plays a pivotal role in anti‐tumor immunity by multiple mechanisms.^89^ Then, the primary tumor sections were stained with H&E, mice treated with PDT+ anti‐PD‐L1 showed more cellular atrophy, necrosis, and chromatin condensation, indicating that this combination therapy had the best antitumor effect (Figure 7j). Furthermore, tumor‐infiltrating lymphocytes (TILs) are an integral component of the tumor microenvironment and have been found to mediate antitumor immune response, especially the CD8^+^ T cell.^91^, ^92^ Immunofluorescence staining revealed that the residual tumors in the control group had no CD8^+^ T cell infiltration (Figure 7k). Interestingly, in the PDT group, there was a large infiltration of CD8^+^ T cells in the superficial area where the tumor was treated with PDT, while there was little inside the tumor. This may be due to light‐induced CD8^+^ T cell migration and infiltration into the tumor, but because the limited depth of light penetration, CD8^+^ T cells are only limited to the superficial layer of the tumor. In the anti‐PD‐L1 group, there were some CD8^+^ T cells infiltrating the tumor. In contrast, those from PDT+ anti‐PD‐L1‐treated tumors were highly infiltrated with CD8^+^ T cells, both superficially and internally in the tumor (Figure 7k). Taken together, these observations suggested that Plt‐M@P‐mediated PDT combined with anti‐PDL‐1 could effectively trigger a robust T‐cell‐mediated anti‐tumor immune response, inhibiting the growth of large‐burden tumors.

### 3.6 Biosafety assessment of combination therapy with PDT mediated by Plt‐M@P and anti‐PD‐L1 immunotherapy

Conventional tumor treatments, including radiotherapy and chemotherapy, often cause serious side effects, such as hypo‐leukemia, liver, and kidney function damage, neuralgia, reproductive toxicity, and so forth.^93^, ^94^ The major side effects of PDT are photosensitivity reactions, including local skin redness, oozing, and hyperpigmentation. To reduce the occurrence of these adverse reactions, reducing the accumulation of photosensitizer at nontumor sites is an effective means. The use of platelet membranes to camouflage the photosensitizer can enhance tumor delivery and reduce its accumulation in normal tissues, thus reducing or avoiding the aforementioned side effects of PDT. In addition, vital organs and functions need to be evaluated to assess the safety of Plt‐M@P‐mediated PDT in combination with anti‐PD‐L1 immunotherapy. H&E staining at the end of the anti‐tumor study revealed no obvious histological damage in the major organs (spleen, liver, lung, heart, and kidney), supporting the biosafety of Plt‐M@P‐mediated PDT combination with anti‐PD‐L1 (Figure S10a). In addition, the level of aspartate transaminase (AST) and alanine aminotransferase (ALT) for liver function, creatinine (CRE) and blood urea nitrogen (BUN) for renal function, and the creatine kinase (CK) and lactic dehydrogenase (LDH) showed no difference between PBS group and treatment groups (Figure S10b). These results further suggested that Plt‐M@P‐mediated PDT and anti‐PD‐L1 did not cause functional changes in major organs. In addition, it is well established that cancer treatment, such as radiotherapy and chemotherapy, can deplete the ovarian reserve, resulting in infertility or early menopause in young women.^95^, ^96^, ^97^ With cancer survivors rapidly growing, there is now a critical need to develop new treatments to address these complications. To explore the reproductive toxicity of Plt‐M@P‐mediated PDT and anti‐PD‐L1, H&E staining of ovary was conducted. The representative histological images of ovaries from four groups exhibited some healthy follicles (Figure S10c, green, primordial follicles; red, primary follicles; yellow, secondary follicles; blue, antral follicles; orange, atretic follicles). The ovarian function is determined by the number of follicles, different levels of follicles were counted. The total number of follicles in the treatment group (PDT, anti‐PD‐L1, PDT+ anti‐PD‐L1) did not differ significantly from that of the PBS group (Figure S10d). In addition, one of the main functions of the ovary is hormone secretion, the concentrations of 17 β‐estradiol (E2) and anti‐Müllerian hormone (AMH) in serum were measured. As shown in Figure S10e, the differences in serum E2 and AMH levels between treatment and PBS groups showed no significance, indicating the photodynamic‐immunotherapy does not damage ovarian function. Taken together, these results suggested that Plt‐M@P‐mediated PDT and anti‐PD‐L1 are safe, and with an almost inexistent toxicity risk to other organs that make it a good candidate for tumor therapy.

## 4 CONCLUSION

In this study, a platelet membrane camouflaged novel AIE photosensitizer, Plt‐M@P, was proposed for antitumor PDT. By taking advantage of the specific affinity between platelets and tumor cells, the Plt‐M@P NPs can efficiently target tumor cell membranes and promote the uptake of the NPs by tumor cells. When applied in small tumor‐bearing mice, the Plt‐M@P NPs were enriched in the tumor and promoted strong ROS generation to induce tumor apoptosis upon light irradiation. While in tumor models with large tumor burdens, the Plt‐M@P and checkpoint inhibitors of anti‐PD‐L1 alone failed to inhibit tumor growth, Plt‐M@P‐mediated PDT combined with anti‐PD‐L1 achieved enhanced antitumor effect. This effect can be attributed to the capability of the photodynamic‐immunotherapy to increase the numbers of immune cells such as CD8^+^ and CD4^+^ T cells. Importantly, this combined therapy had no damage to the main organs and especially the reproductive toxicity of traditional tumor therapy can be avoided. These outcomes might provide an alternative nanomedicine for efficient therapy of tumors with large burdens. However, to produce the amount of Plt‐M@P needed to acquire the ideal therapeutic effect, relatively large numbers of platelet are required. Hence, moving forward, the low yield of membrane extraction should be improved. In conclusion, with the modification of the isolation and purification processes, these biomimetic NPs may represent a safe therapeutic platform with promising high therapeutic efficiency for clinical cancer treatment.

## AUTHOR CONTRIBUTIONS

**Jun Dai:** Data curation (lead); formal analysis (lead); funding acquisition (equal); methodology (lead); writing – original draft (lead); writing – review and editing (lead). **Meng Wu:** Funding acquisition (equal); investigation (equal); methodology (equal); software (equal); visualization (equal); writing – original draft (equal). **Yating Xu:** Investigation (equal); methodology (equal); validation (equal); writing – original draft (equal). **Hongming Yao:** Methodology (supporting); writing – original draft (supporting). **Xiaoding Lou:** Formal analysis (equal); investigation (equal); supervision (equal); writing – original draft (equal). **Yuning Hong:** Visualization (equal); writing – original draft (equal). **Shixuan Wang:** Conceptualization (lead); funding acquisition (lead); project administration (lead); resources (lead); supervision (lead).

### PEER REVIEW

The peer review history for this article is available at https://publons.com/publon/10.1002/btm2.10417.

## Supporting information


**APPENDIX S1** Supporting Information

## Data Availability

The data that support the findings of this study are available from the corresponding author upon reasonable request.
