# Supplementary material for: Platelet membrane camouflaged AIEgen‐mediated photodynamic therapy improves the effectiveness of anti‐PD‐L1 immunotherapy in large‐burden tumors
Source: Bioeng Transl Med. 2022 Oct 6;8(2):e10417. doi: 10.1002/btm2.10417 (PMC10013814; doi:10.1002/btm2.10417)
Supplement: Supplementary file 1 — APPENDIX S1 Supporting Information [file BTM2-8-e10417-s001.docx]

Supporting Information

**Platelet Membrane Camouflaged AIEgen-Mediated Photodynamic Therapy Improves the Effectiveness of Anti-PD-L1 Immunotherapy in Large-Burden Tumors**

Jun Dai,^†a^ Meng Wu,^†a^ Yating Xu,^†b^ Hongming Yao,^b^ Xiaoding Lou,^c^ Yuning Hong,^d^ Jian Zhou,^*b^ Fan Xia, ^*c^ Shixuan Wang,^*a^

^a^ Department of Obstetrics and Gynecology, Tongji Hospital, Tongji Medical College, Huazhong University of Science and Technology, Wuhan 430074, China. Email: shixuanwang@tjh.tjmu.edu.cn

^b^ College of Material, Chemistry and Chemical Engineering, Hangzhou Normal University, Hangzhou 310036, China. Email: zhoujian@hznu.edu.cn

^c^ State Key Laboratory of Luminescent Materials and Devices, Guangdong Provincial Key Laboratory of Luminescence from Molecular Aggregates, South China University of Technology, Guangzhou 510640, China

^d^ State Key Laboratory of Biogeology and Environmental Geology, Engineering Research Center of Nano-Geomaterials of Ministry of Education, Faculty of Materials Science and Chemistry, China University of Geosciences, Wuhan 430074, China. Email: xiafan@cug.edu.cn

^†^ These authors contribute equally to this work.


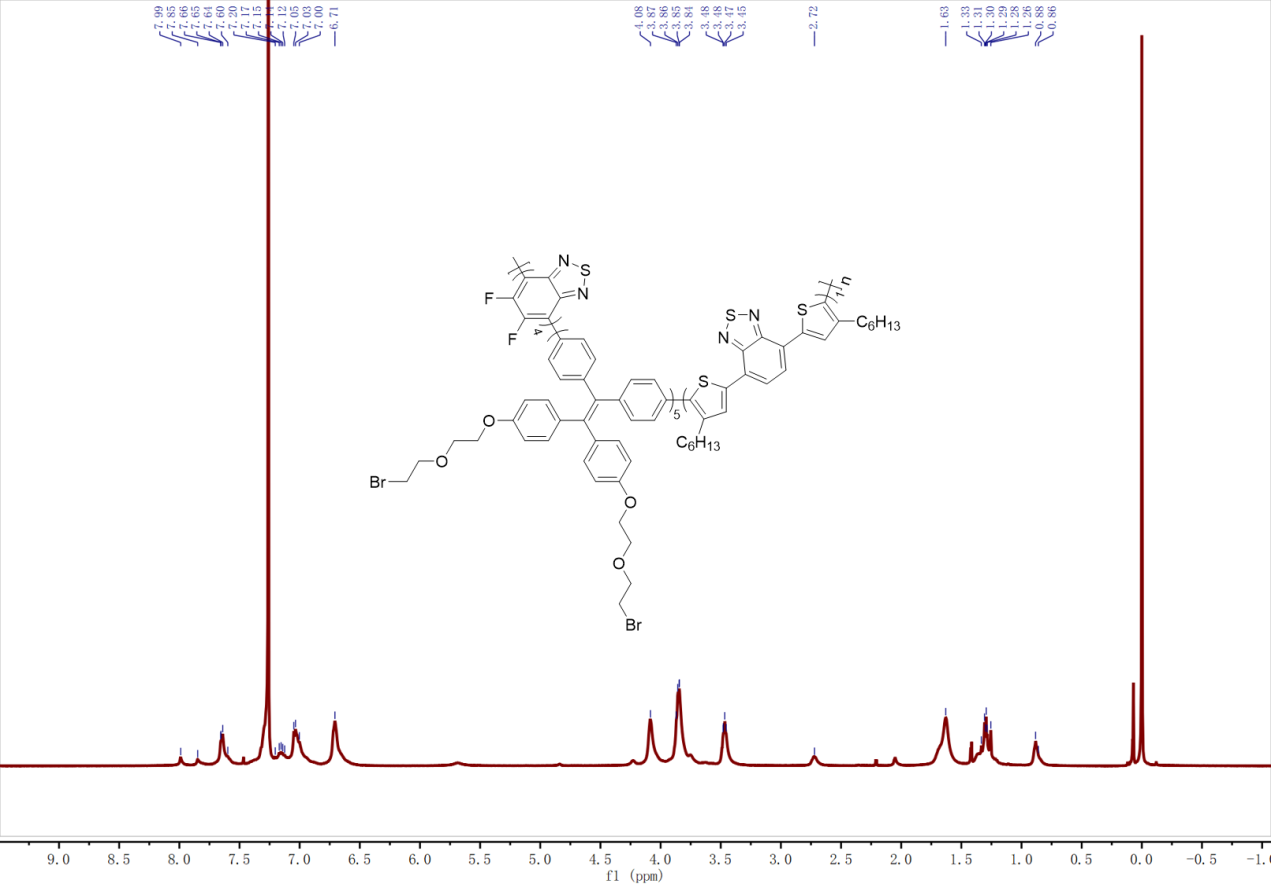


**Figure S1**. Hydrogen spectrum of PF3


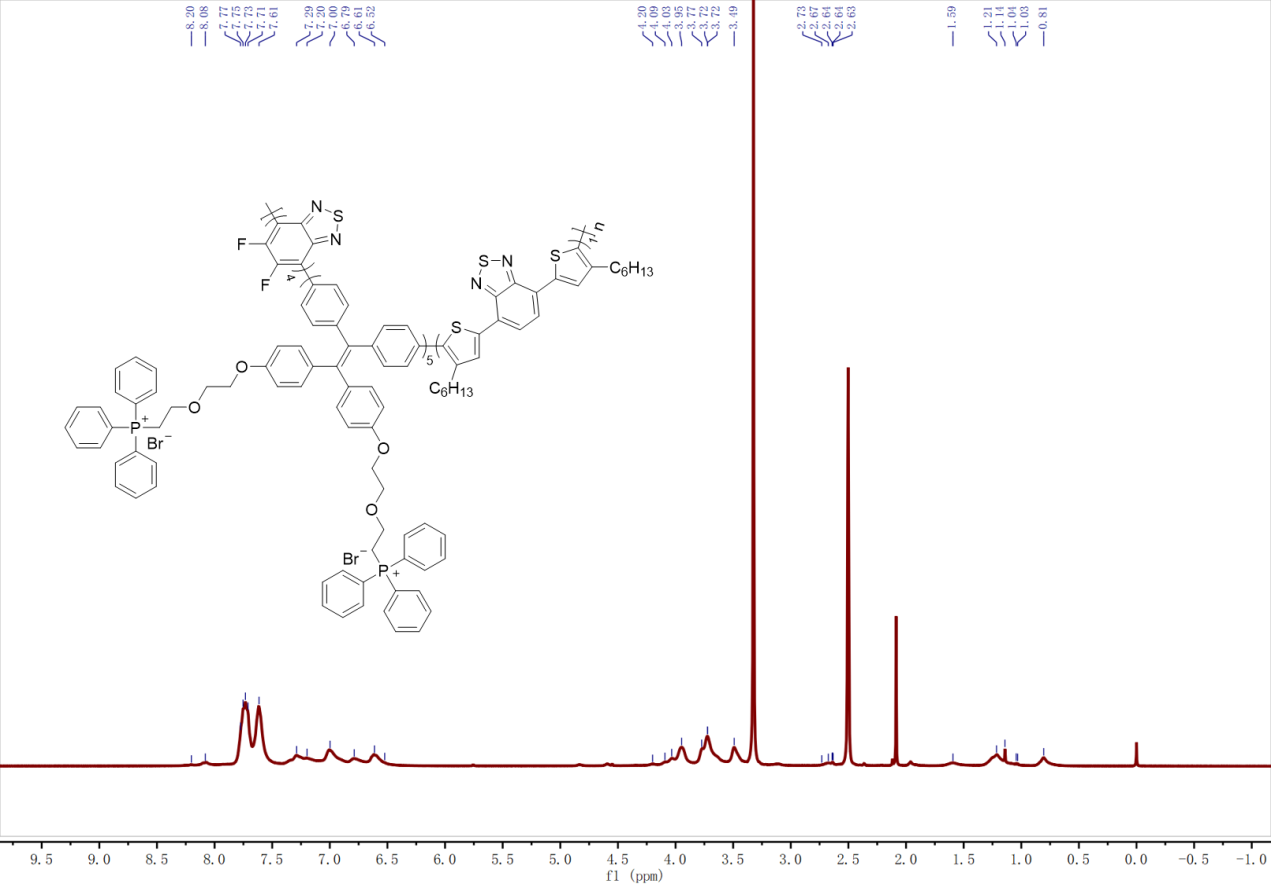


**Figure S2**. Hydrogen spectrum of PF3-PPh_3_


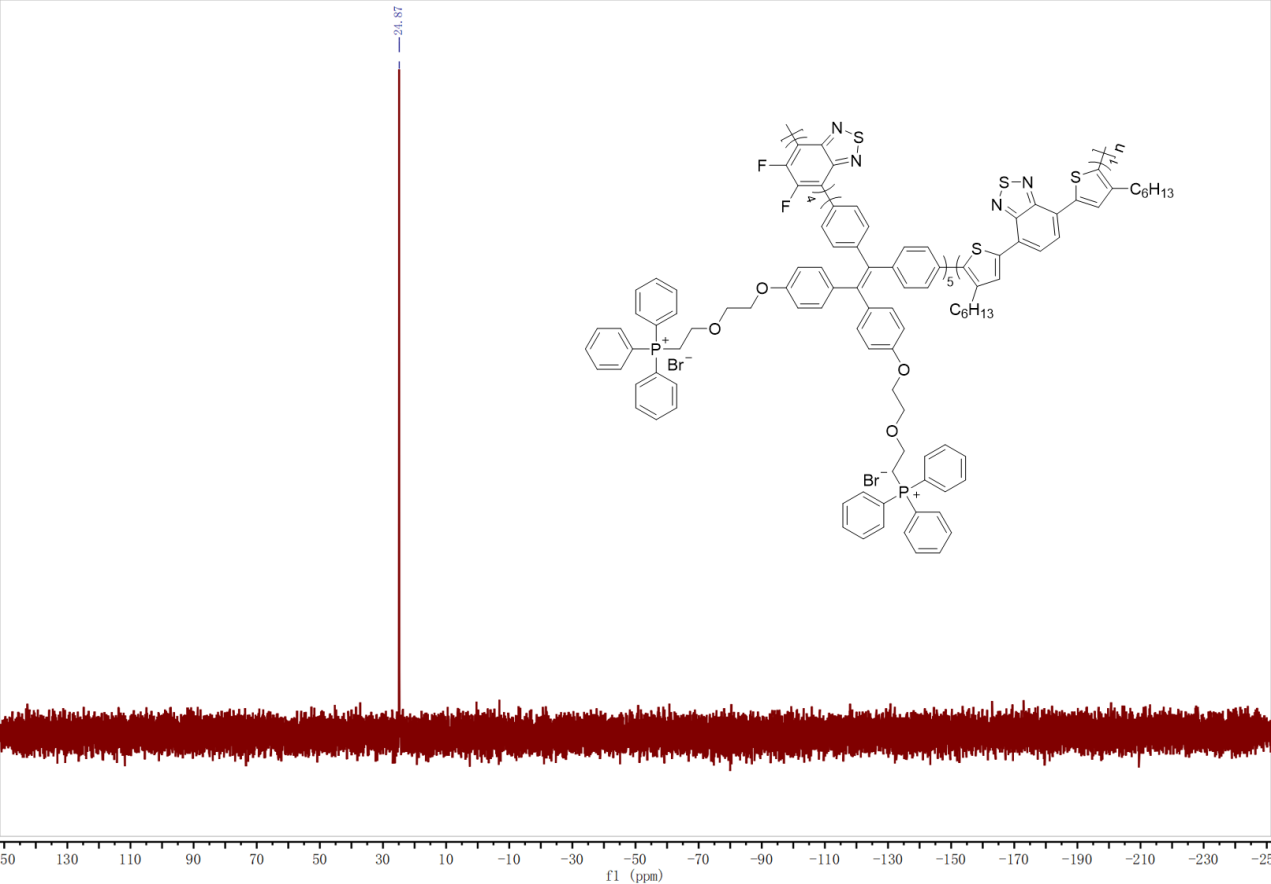


**Figure S3**. Phosphorus spectrum of PF3-PPh_3_


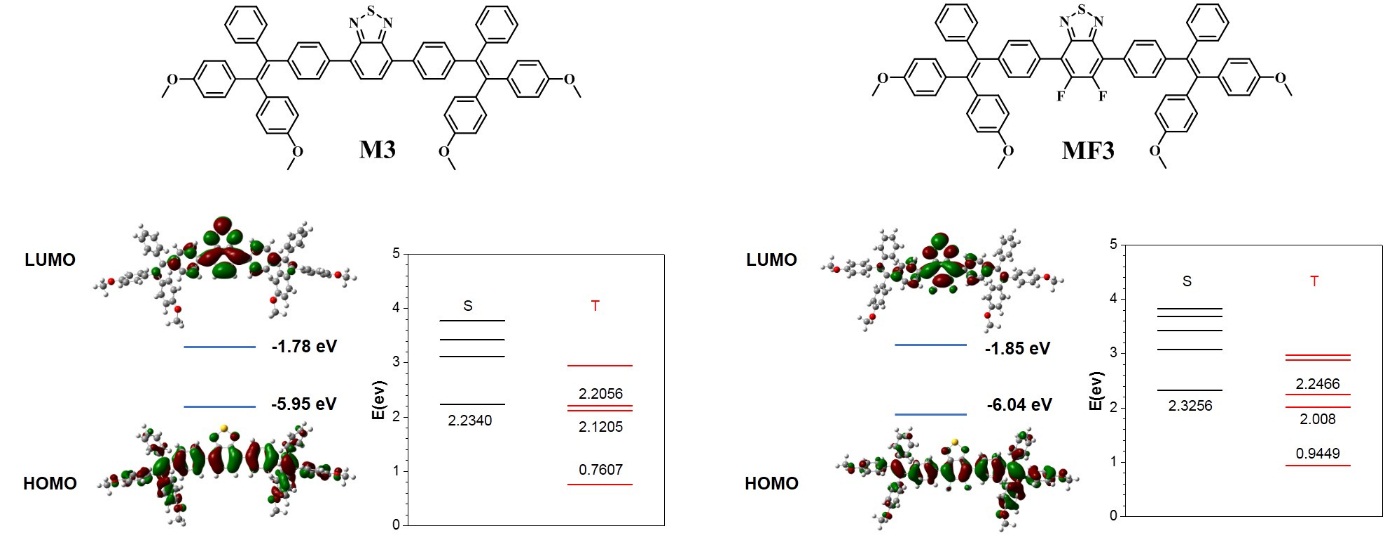


**Figure S4**. The molecular structure of the conjugated fragments M3 of P3-PPh_3_ and MF3 of PF3-PPh_3_. The HOMO and LUMO distributions of M3 and MF3 calculated by DFT at B3LYP/6-31G* method, energy levels of S and T obtained by TD-DFT on B3LYP/6-311G** method.


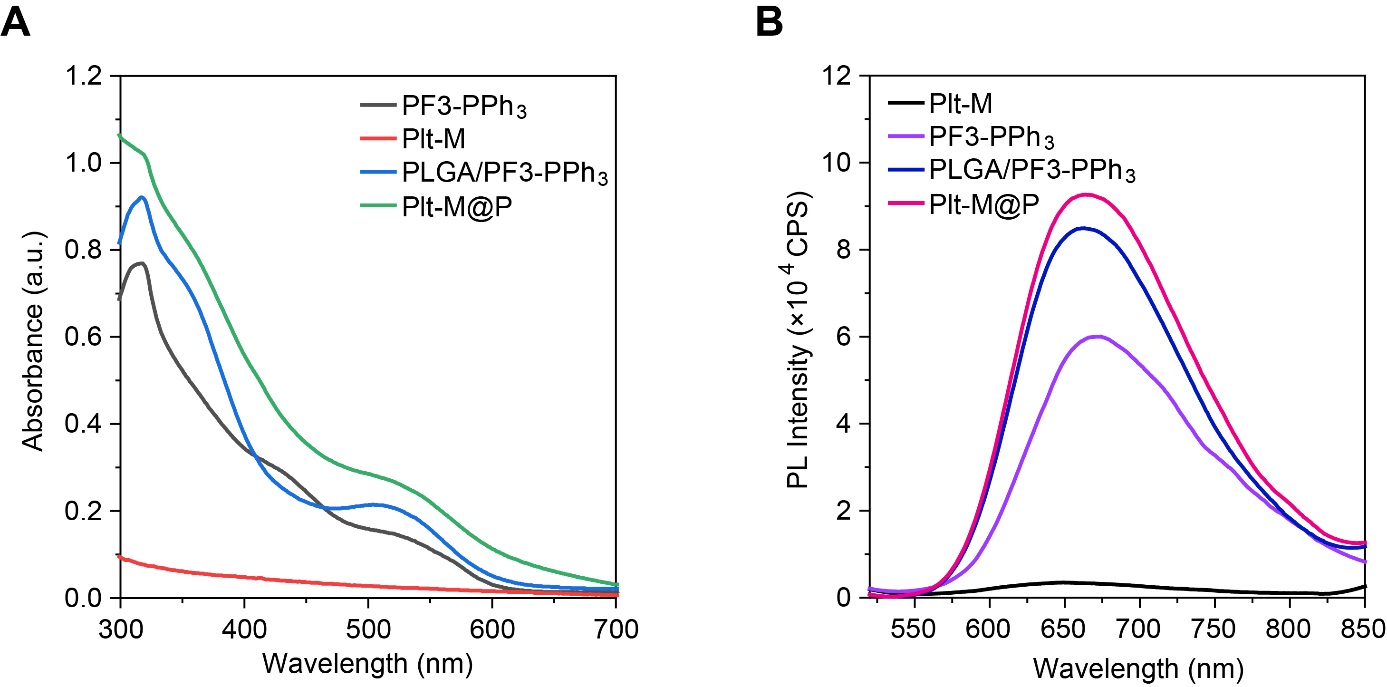


**Figure S5**. (A) UV-Vis spectra of PF3-PPh_3_, Plt-M, PLGA/PF3-PPh_3_ and Plt-M@P. (B) PL spectra (Ex =506 nm) of PF3-PPh_3_, Plt-M, PLGA/PF3-PPh_3_ and Plt-M@P.


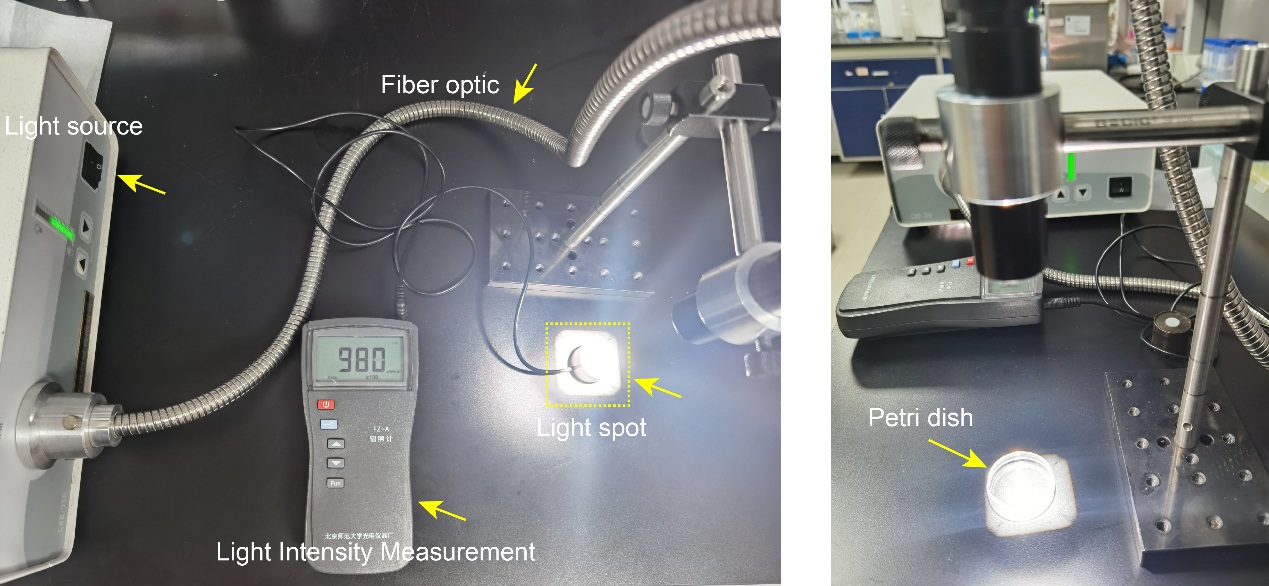


**Figure S6**. Equipment involved in photodynamic systems, and their use for the detection of ROS in solution.


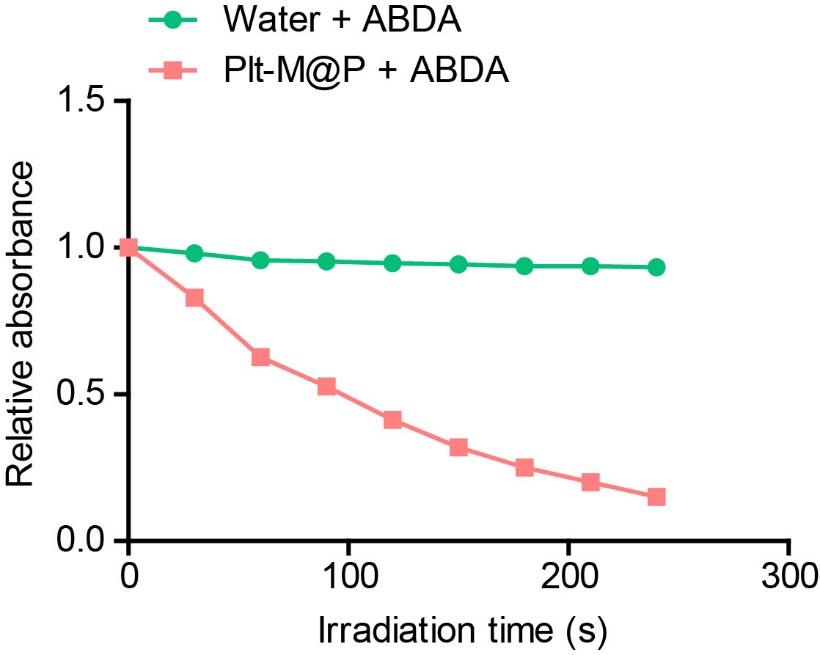


**Figure S7**. Quantitative analysis of the amount of ROS produced in water and Plt-M@P. The concentration of ABDA is 50 μM. The concentration of PF3-PPh_3_ is 10 μg/mL. The white light intensity is 100 mW cm^-2^.


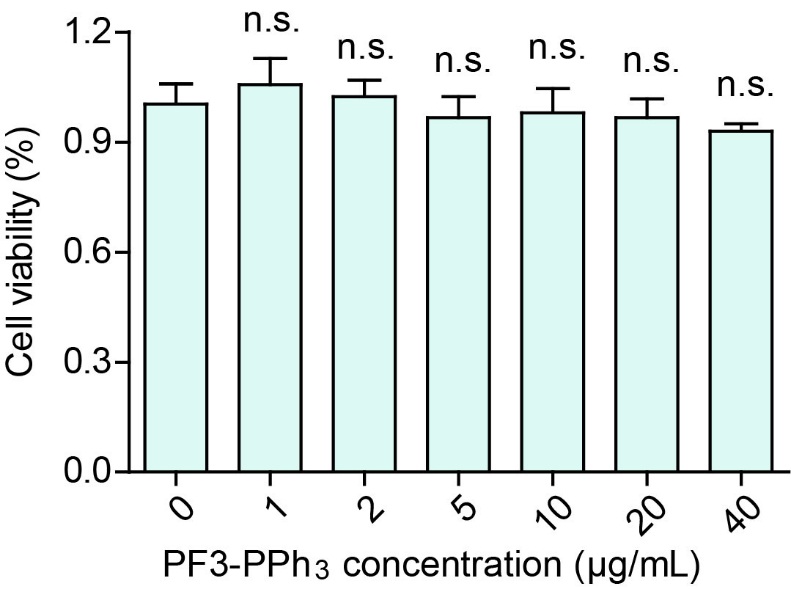


**Figure S8**. Viability of HLF cells treated with different concentration of Plt-M@P without light irradiation. The data were reported as mean ± SD and analyzed by two-sided Student’s t-test. n.s. not significant.


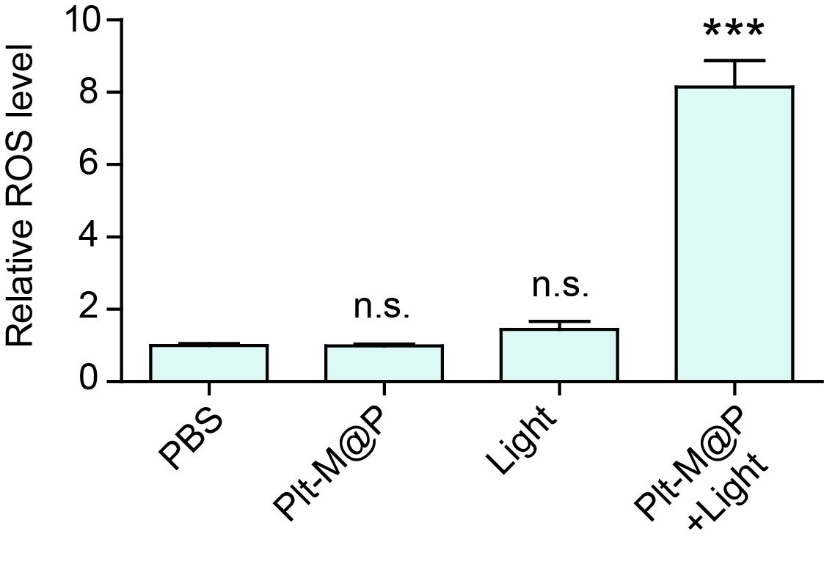


**Figure S9**. Quantitative analysis of ROS levels in 4T1 cells treated with different treatments. The data were reported as mean ± SD and analyzed by two-sided Student’s t-test. *** *p*< 0.001, n.s. not significant.

**
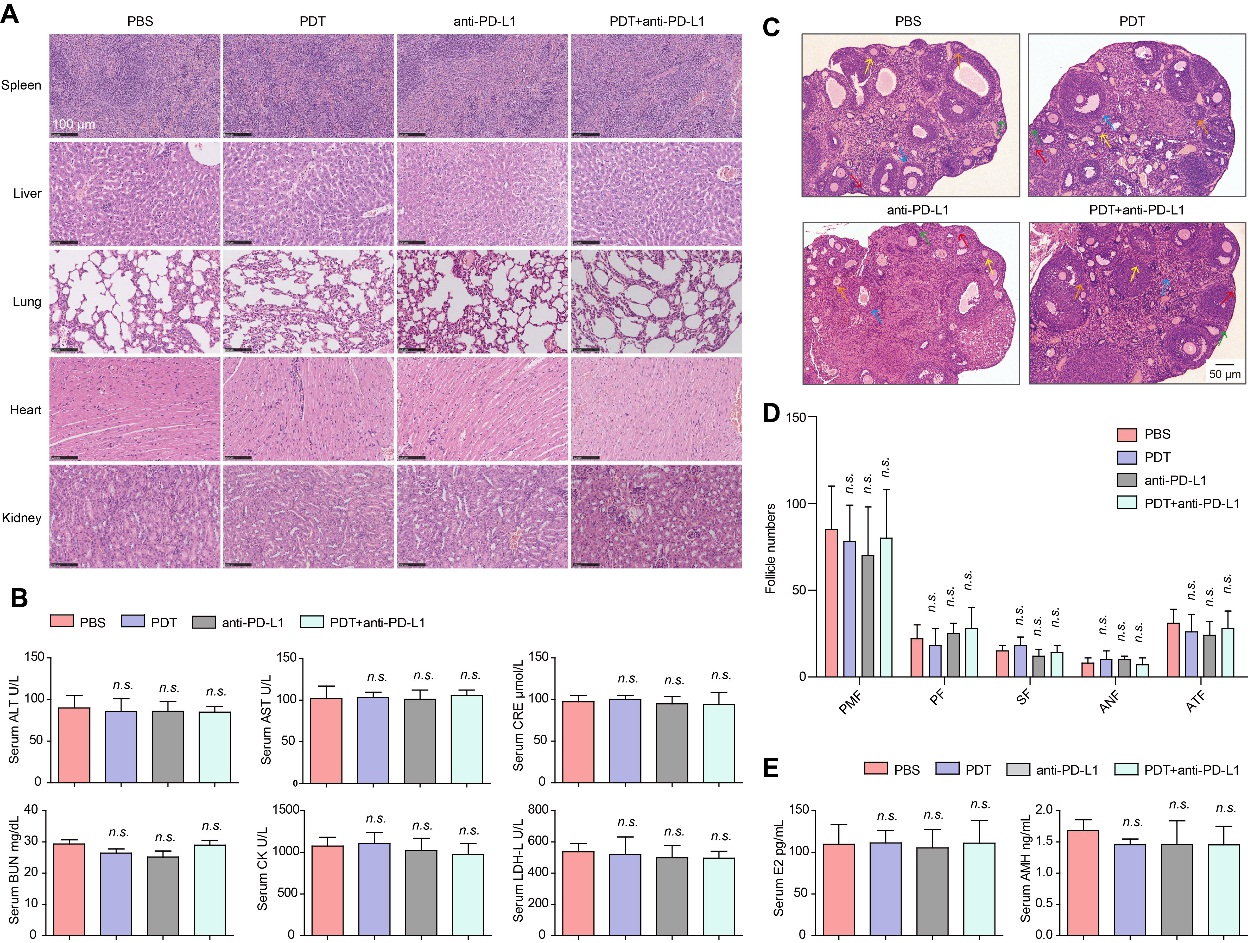
**

**Figure S10**. Biosafety assessment of PDT and anti-PD-L1 combination therapy. (A) H&E staining images of major organs (spleen, liver, lung, heart, and kidney) of bearing 4T1 tumor mice in control and treatment groups. Scale bar =100 µm. (B) The level of AST and ALT for liver function, CRE and BUN for renal function, and the CK and LDH-L for heart function. (C) H&E staining images of ovaries from four groups. Green arrow: primordial follicles; Red arrow: primary follicles; Yellow arrow: secondary follicles; Blue arrow: antral follicles; Orange arrow: atretic follicles. Scale bar =50 μm. (D) Follicle number and proportion in different groups. PMF: primordial follicle, PF: primary follicle, SF: secondary follicle, ANF: antral follicle, ATF: atretic follicle. (E) Levels of serum E2 and AMH in mice from four groups. The data were reported as mean ± SD and analyzed by two-sided Student’s t-test. n.s. not significant.
